# Molecular determinants for the thermodynamic and functional divergence of uniporter GLUT1 and proton symporter XylE

**DOI:** 10.1371/journal.pcbi.1005603

**Published:** 2017-06-15

**Authors:** Meng Ke, Yafei Yuan, Xin Jiang, Nieng Yan, Haipeng Gong

**Affiliations:** 1 MOE Key Laboratory of Bioinformatics, Tsinghua University, Beijing, China; 2 State Key Laboratory of Bio-membrane and Membrane Biotechnology, Tsinghua University, Beijing, China; 3 Beijing Advanced Innovation Center for Structural Biology, Tsinghua University, Beijing, China; 4 Tsinghua-Peking Center for Life Sciences, School of Life Sciences and School of Medicine, Tsinghua University, Beijing, China; Max Planck Institute for Biophysical Chemistry, GERMANY

## Abstract

GLUT1 facilitates the down-gradient translocation of D-glucose across cell membrane in mammals. XylE, an *Escherichia coli* homolog of GLUT1, utilizes proton gradient as an energy source to drive uphill D-xylose transport. Previous studies of XylE and GLUT1 suggest that the variation between an acidic residue (Asp27 in XylE) and a neutral one (Asn29 in GLUT1) is a key element for their mechanistic divergence. In this work, we combined computational and biochemical approaches to investigate the mechanism of proton coupling by XylE and the functional divergence between GLUT1 and XylE. Using molecular dynamics simulations, we evaluated the free energy profiles of the transition between inward- and outward-facing conformations for the *apo* proteins. Our results revealed the correlation between the protonation state and conformational preference in XylE, which is supported by the crystal structures. In addition, our simulations suggested a thermodynamic difference between XylE and GLUT1 that cannot be explained by the single residue variation at the protonation site. To understand the molecular basis, we applied Bayesian network models to analyze the alteration in the architecture of the hydrogen bond networks during conformational transition. The models and subsequent experimental validation suggest that multiple residue substitutions are required to produce the thermodynamic and functional distinction between XylE and GLUT1. Despite the lack of simulation studies with substrates, these computational and biochemical characterizations provide unprecedented insight into the mechanistic difference between proton symporters and uniporters.

## Author summary

We seek to address one intriguing question, the mechanistic distinction between active proton-coupled symporters and passive uniporters that are related in evolution. Proton-coupled symporters harness the transmembrane proton gradient to drive the substrate transport, while uniporters can only facilitate the passive substrate translocation. In this work, we focus on two sugar transporters GLUT1 and XylE, which belong to symporters and uniporters respectively but have high sequence similarity. We first applied molecular dynamics simulations to characterize the thermodynamic behaviors of *apo* GLUT1 and XylE, which are supposed to provide prominent details of mechanisms. From the identified difference in thermodynamics, we concluded that neutralizing protonation site in XylE is insufficient for its conversion to GLUT1 analog. To pinpoint extra elements contributing to their evolutionary divergence, we developed a novel network modeling scheme based on Bayesian network which shows impressive predictive power on residue mutations. Our models suggested the detailed mechanism of proton coupling in XylE and molecular basis of symporter/uniporter discrepancy. Furthermore, the modeling scheme could help to guide the design of biomolecules for desired functions.

## Introduction

The glucose transporter GLUT1 catalyzes facilitative diffusion of glucose into red blood cells[1] and across the blood-brain barrier[2]. The bacterial homologues of GLUT1 are all proton symporters whereby the transmembrane proton gradient is employed to drive the uphill translocation of the substrate saccharides into the cell[3]. The distinct transport mechanisms are consistent with their working environment. The glucose concentration in blood maintains at around 5 mM and the intake glucose is immediately metabolized to glucose-6-phosphate in the cytosol, thereby creating a constant transmembrane gradient of glucose[4]. A facilitative uniporter is thus sufficient to mediate the uptake of glucose. In contrast, bacteria may have to hunt under stringent conditions. A co-transport mechanism may ensure efficient uptake of the nutrient at low concentration in the environment. Interestingly, GLUT1 and its bacterial homologues share considerable sequence similarities[5], raising the question of what is the determinant under the mechanistic divergence between the closely related proton symporter vs. uniporter.

The xylose:proton symporter XylE from *E*. *coli* is one of a number of rigorously characterized GLUT1 homologues. In recent years, crystal structures of GLUTs[6,7,8] and XylE were determined in multiple conformational states[5,9,10], in line with alternating access mechanism of membrane transporters[11]. Structure-guided mutational analysis identified Asp27 of XylE to be the protonation site for symport[12]. The D27N variant of XylE that was designed to mimic the neutral residue Asn29 at the corresponding position of GLUT1, however, lost transport activity in *in vivo* experiments, despite fully active in *in vitro* counter-flow assays[12]. Thus, the behavioral difference between uniporters and symporters exemplified by GLUT1 and XylE cannot be accounted for simply from the perspective of protonation-site residues. Instead, atomic-level description on the transitions between alternating-access states and quantitative evaluation on the thermodynamics of these processes are required to address these questions.

Molecular dynamics (MD) simulations, which resemble *in silico* single-molecule experiments at atomic resolution, emerge as a suitable tool for investigating conformational transitions of macromolecules[13]. In addition to the direct observation on large-scale conformational changes, thermodynamics and many physical properties could be rigorously evaluated to elucidate the internal causes of molecular behaviors[14,15,16,17]. In this work, we used MD simulations to study the alternating-access transitions of three *apo* systems: XylE with Asp27 protonated (denoted as XylE_H), XylE with Asp27 deprotonated (denoted as XylE_noH) and GLUT1. Despite the lack of substrates, these simulations provided the quantitative details for the structural transitions from the inward-facing (IF) to outward-facing (OF) states, which are informative parts of the complete transport cycle. From the free energy profiles calculated for the transitions, we not only revealed the coupling between Asp27 protonation/deprotonation and conformational transition of XylE, but also identified a remarkable thermodynamic difference between XylE and GLUT1. Subsequently, to further understand the mechanism of these thermodynamic observations, we developed Bayesian network (BN) models to analyze changes of residue interaction networks during conformational transitions. Besides mechanistic illustration, these statistical models predicted a few residues essential for the appropriate conformational preference in XylE, which was then validated by experiments on the corresponding mutants. More importantly, our results suggested that the thermodynamic divergence between XylE and GLUT1 arises from multiple residue substitutions accumulated during evolution. Based on a group of residues inferred from our models, we successfully designed a uniporter-like XylE mutant, which was then confirmed by experimental validation. Conclusively, our computation results provided insight into the mechanistic difference between proton symporters and uniporters in the absence of substrates.

## Results

### Conformation sampling and free energy estimation

GLUTs and XylE have a classical MFS fold of 12 transmembrane (TM) helices plus a unique intracellular helical (ICH) domain that comprises of 4 or 5 helices[7]. Structural comparison and accompanying biochemical characterizations of the sugar porter (SP) family members suggested that the two TM domains, the N-terminal domain (NTD) and C-terminal domain (CTD), undergo concentric rotations relative to each other to accomplish transition between inward- and outward-facing conformations, resulting in the alternating access of the substrate binding site(s) from either side of the membrane[18,19].

**Fig 1:**
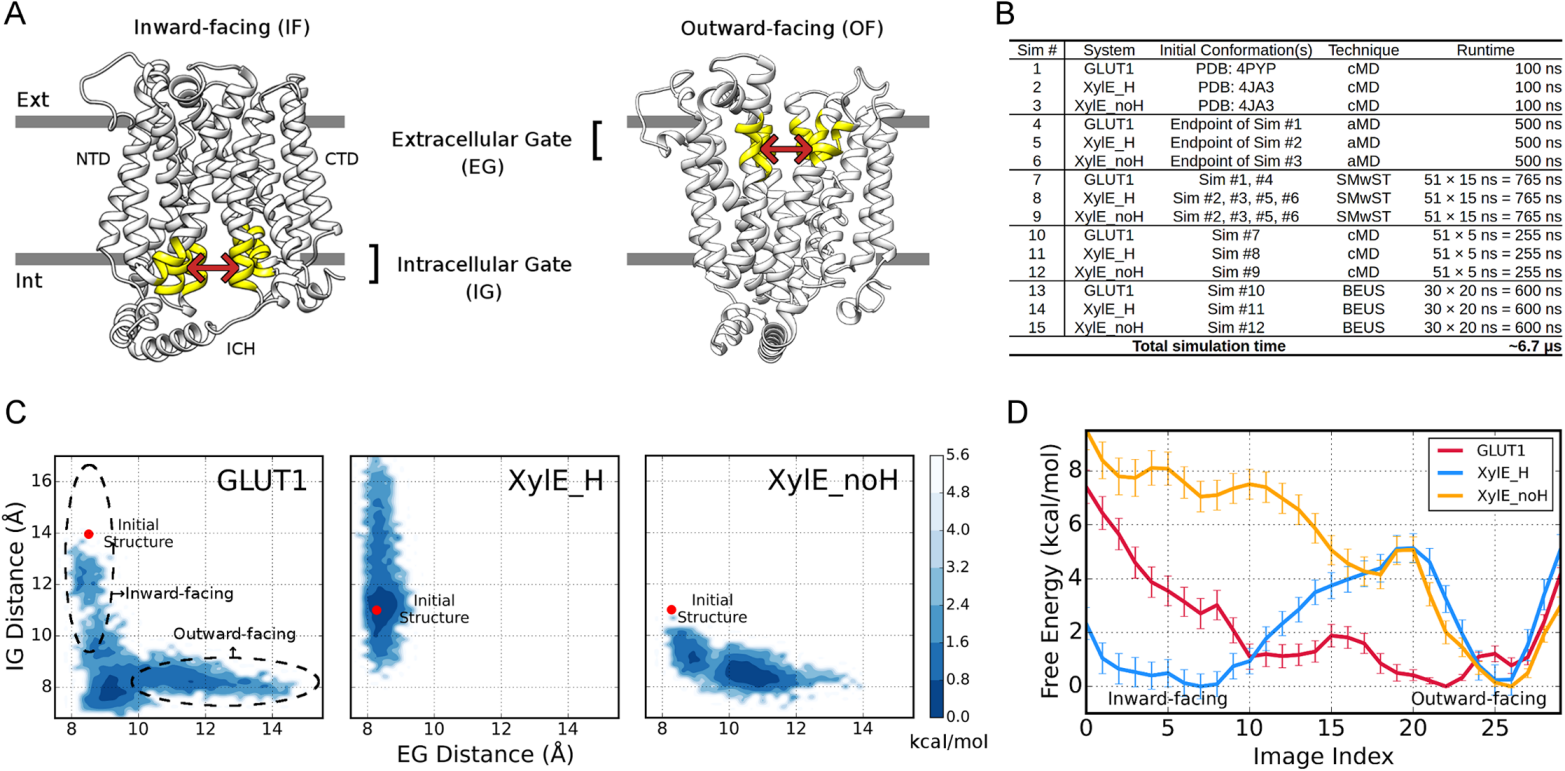
Conformation sampling and free energies of GLUT1, XylE_H and XylE_noH. **(A)** The definitions of extracellular and intracellular gates. Extracellular gate (EG) comprises of two groups of interacting helices at periplasmic side, including TM 1, 2&5 and TM 7, 8&11 (highlighted in yellow). Likewise, cytoplasmic portions of TM 2, 4&5 and TM 8, 10&11 compose the intracellular gate (IG) residues. The centers of mass (COM) of Cα atoms in the two gating groups are used to depict gate distances. **(B)** List of simulation sets performed on GLUT1, XylE_H and XylE_noH systems. **(C)** The unweighted contour maps for aMD trajectories in the 2D space of Extracellular and Intracellular Gate distances. The positions of initial structures for GLUT1 (PDB ID: 4PYP) and XylE systems (PDB ID: 4JA3) are shown as red dots. Dashed circles denote the supposed regions of inward- and outward-facing conformations. **(D)** Free energy profiles along discretized BEUS images/windows. GLUT1, XylE_H and XylE_noH are colored red, blue and yellow, respectively.

To study the conformational transition, we started from the inward open GLUT1 and inward occluded XylE with Asp27 protonated/deprotonated denoted as XylE_H/XylE_noH hereafter. The conventional MD (cMD) simulations (Sim #1, #2 and #3 in Fig 1B) revealed little change with RMSDs of TM domains < 1.5 Å (S1A Fig) for all three systems. Subsequently, accelerated MD (aMD) simulations were initiated to encourage conformational transitions without directional inclinations (Sim #4, #5 and #6). Conformational states captured by aMD simulations were illustrated on a 2D-map of Extracellular Gate distance and Intracellular Gate distance between NTD and CTD, which depict the extent of opening towards periplasm and cytoplasm respectively (Fig 1A, see Free energy calculations in Methods section). As shown in Fig 1C, GLUT1 rapidly left the initial state and underwent IF→OF transition (also in S1B Fig), suggesting that the uniporter has a barrier-free energy landscape consistent with facilitative transport. Unlike GLUT1, aMD trajectories of XylE_H and XylE_noH were confined to inward- and outward-facing conformations respectively, without discernible IF↔OF transitions (Fig 1C; S1C and S1D Fig). Therefore, *apo* XylE might possess high-energy transition state(s) that would require substrate binding to stabilize.

We then performed extensive string method with swarms of trajectories (SMwST) simulations using conformations selected from the aMD and cMD trajectories for transition path identification (Sim #7, #8 and #9; see S2B Fig for convergence). Further equilibrations of on-path images (Sim #10, #11 and #12) permitted path reconstruction in the space of gate distances and subsequent free energy calculations by bias-exchange umbrella sampling (BEUS) scheme (S2A Fig). In the BEUS simulations (Sim #13, #14 and #15), each window/image represents a conformation of the IF→OF transition pathway. The final free energies were evaluated (see Free energy calculations in Methods section) both in the space of gate distances (S2C Fig) and along window/image index (Fig 1D). Low RMSDs between structures in individual windows and the crystal structures of various states validate the efficacy of our path-finding protocol (S1E Fig).

The three systems exhibit drastically different free energy profiles (Fig 1D). GLUT1 shows a broad energy well which is required for the fast turnover of passive uniporters. The slight preference of OF over IF conformations agrees with the unidirectional conformational shift in the aMD simulation (S1B Fig). Notably, the GLUT1 profile exhibits no favorable energy wells at the window corresponding to the inward-facing crystal structure 4PYP (S1F Fig). The reason at least partially arises from the mutation and detergent introduced to the crystal structure (see S1 Text). In contrast, an energy barrier is present at the image index of 20 in both XylE systems. According to our rough estimation, this barrier of ~5 kcal/mol can effectively forestall rapid IF↔OF transition (Fig 1D) and thereby suppressing proton leakage across the membrane in the absence of substrate (see S1 Text for details). The marked difference between XylE_H and GLUT1 at the energy barrier cannot be accounted for purely by the protonation-site residue. In addition, comparison on free energy profiles between XylE_H and XylE_noH indicates that conformational preference of XylE could be altered thermodynamically by protonation/deprotonation. Particularly, once the proton and substrate have been unloaded, inward-facing XylE would spontaneously restore the energetically more stable outward-facing state for another cycle of transport.

The systematic difference between XylE and GLUT1 may hinder accurate side-by-side comparison on their free energy profiles. We thus developed an RMSD-scoring based algorithm to align BEUS windows for the three systems, which can effectively eliminate the cross-system window shift along the path (see Window alignment in Methods section; S3 Fig). From the post-alignment free energy profiles of the three systems, the IF and OF states as well as the transition state (TS) can be unanimously represented by windows renumbered as A3, A16 and A12 respectively (S2C Fig and S3C Fig).

### Global and local conformational changes

Comparing aligned states of the three systems, the most noteworthy motion that can be visualized during transition is the domain rotation (S1 Movie and S4B Fig). Hence, we superimposed all structures upon their NTDs and investigated the global conformational changes using principal component analysis (PCA) on TM helices (see Analysis techniques in Methods section). The top 3 principal components explain ~85% of the structural variations in all systems and show strong inter-system correlations, which reflects consensus rocker-switch movements for both uniporters and symporters (S4 Fig). Local changes accompanying the conformational transition were characterized by monitoring the per-residue structural fluctuations among all aligned states (S5 Fig). As expected, NTD and CTD remain nearly rigid in majority of the TM helices (RMSF < 1 Å). In addition to the loop regions, TM7b exhibits flexibility within all three systems, and thus may be a local gating element coupled with the global conformational transition.

The TMs show different packing patterns in the extracellular and intracellular gates. Unlike side-by-side helix anchoring in the former, TM5 and TM11 insert into the interfaces between TM8/10 and TM2/4 by partial twisting in the latter (S4C Fig). The relationship between global conformational transition and gating can be seen from the pore radius analysis (S6C Fig). Interestingly, we identified a unique gate around Tyr298 in XylE that confines the periplasmic entrance (pointed by arrows in S6C Fig). Although this gate becomes fully open in a transient state (window A18) to allow substrate binding, its constriction tendency may cause considerably lower rate of substrate dissociation in XylE than in GLUT1. Apart from the global movement, TM7b bending contributes to the extracellular gating (see the definition of kinking angle in S6A Fig). In GLUT1, the severe kinking of TM7b diminishes in the outward-facing state that exhibits striking structural resemblance to the outward-open GLUT3 (PDB ID: 4ZWC). Conversely, the helix remains bended in the outward-facing conformations of both XylE_H and XylE_noH, thus creating the unique gate (S6 Fig). A proline (Pro301) residing in the middle of TM7b indicates why this helix is reluctant to adopt the straight conformation in XylE.

**Fig 2:**
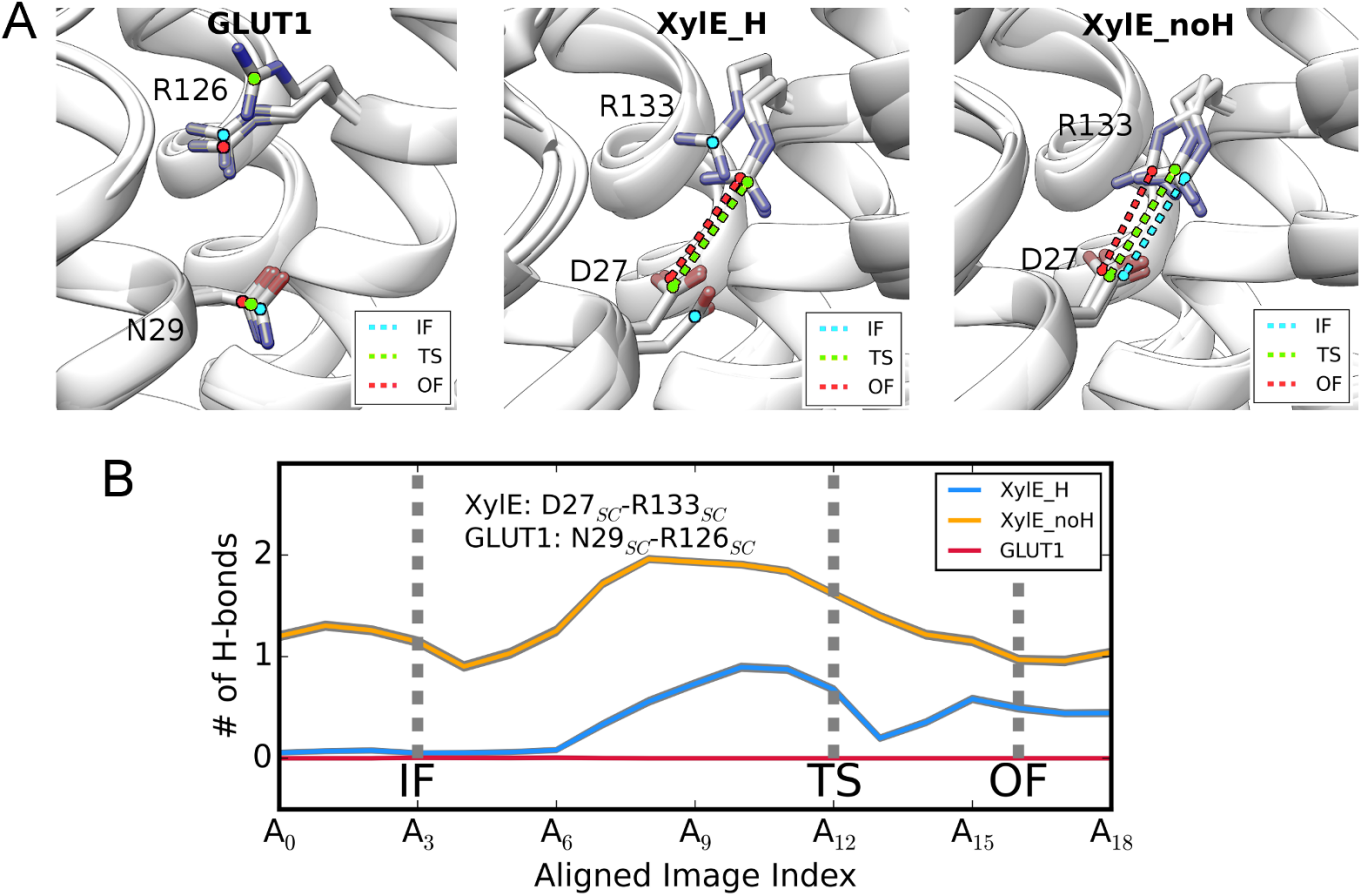
Local changes around protonation site. **(A)** The Asp27_SC_-Arg133_SC_/Gln29_SC_-Arg126_SC_ interaction of XylE/GLUT1 in various conformational states. The interaction is highlighted as dashed lines only when the two interacting partners form close physical contact. The interaction is labeled in cyan, green and red for IF, TS and OF states, respectively. **(B)** The average number of H-bonds formed between the Asp27_SC_-Arg133_SC_/Gln29_SC_-Arg126_SC_ interacting pairs along the aligned transition paths of GLUT1 (red), XylE_H (blue) and XylE_noH (yellow). The gray shadow around each curve represents standard deviation.

The above conformational changes cannot explain the functional and thermodynamic differences between uniporter GLUT1 and symporter XylE despite that they are consistent with the structural studies. We then switched our focus to the interactions around the meaningful protonation-site residue, i.e. Asp27-Arg133 bonding in XylE and the corresponding Asn29-Arg126 interaction in GLUT1, as reported[5,10]. Analysis on hydrogen bonds (H-bonds) reveals that side-chains of the Asn29-Arg126 pair in GLUT1 never form favorable interactions in any conformational states, whereas those of Asp27-Arg133 in XylE_H preserve moderate H-bonds in TS and OF states (Fig 2; S1 Movie). This observation and thermodynamic distinction between GLUT1 and XylE_H jointly negate the proposition that simple neutralization of Asp27 in XylE could eliminate their functional gap. The Asp27-Arg133 bonding in XylE_noH system is well maintained in all conformations due to electrostatic attraction, which is consistent with structural observations[10]. In contrast, this interaction is greatly impaired in the inward-facing state of XylE_H. Considering that XylE_H and XylE_noH only differs in the protonation state of Asp27, changed bonding strength of Asp27-Arg133 could be the origin of their different thermodynamics.

### The interaction networks for different conformational states

To understand the molecular basis for the thermodynamic difference between XylE and GLUT1 observed in simulations, we made side-by-side comparison on the changes in residue interaction networks during their conformational transitions. Conventional modeling strategies of residue interaction network usually construct undirected graphs with nodes representing residues or Cα atoms, and with edges reflecting correlated motions and/or physical contacts[20]. Although it has been reported that causality could be extracted from such networks[21], these mutual-information based approaches show limited predictive power on network tuning and had a poor performance in our case.

**Fig 3:**
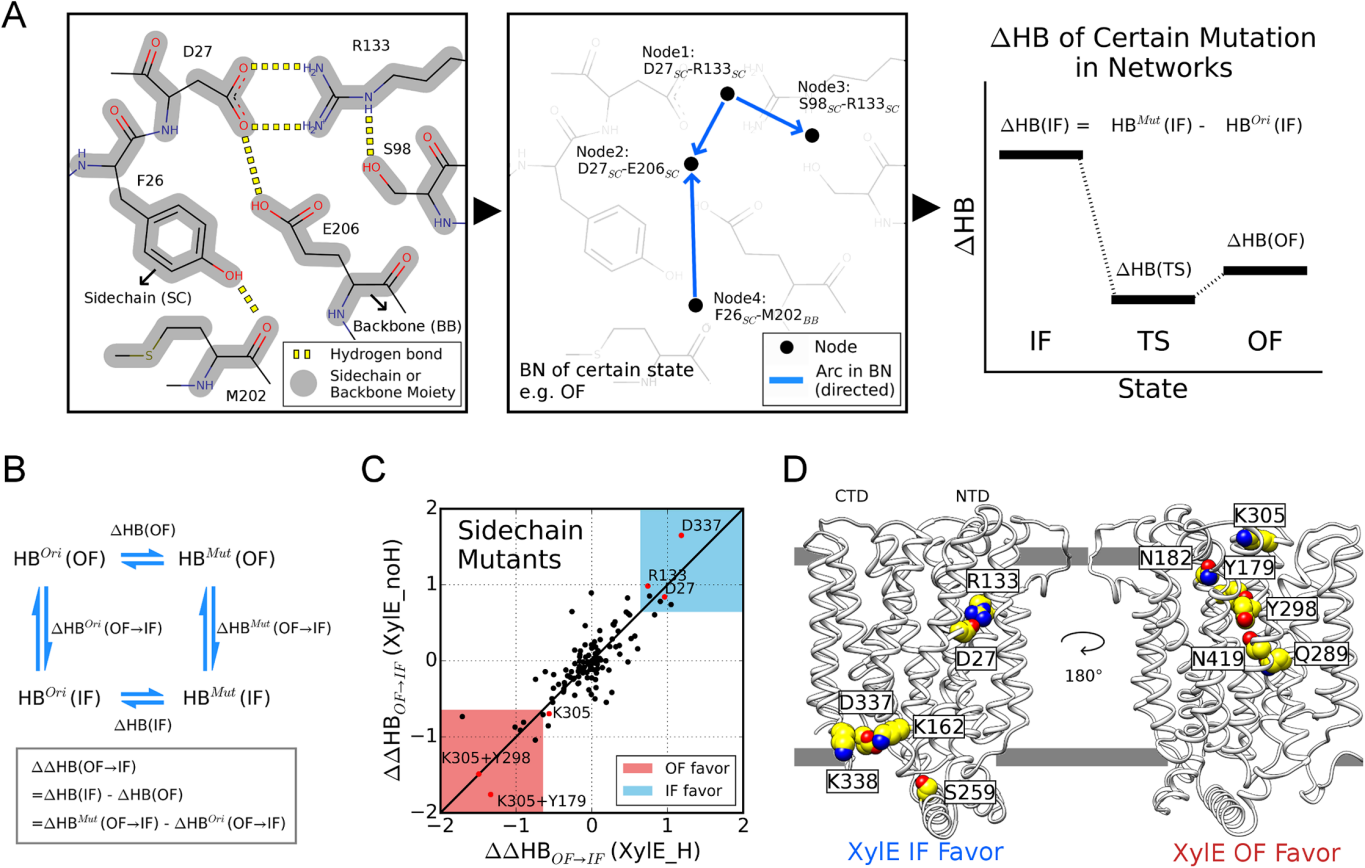
Modeling of causal networks for H-bonds. **(A)** Schematic diagram of Bayesian network learning and inference (see S7 Fig for details). (***Left panel***) Non-trivial H-bonds (yellow) are identified for a particular conformation. (***Middle panel***) A network model is constructed by Bayesian network learning, where H-bonds are abstracted as nodes (black dots) and their causal relationships are represented as directed arcs. (***Right panel***) In Bayesian network inference, the change in the total amount of H-bond (ΔHB) caused by certain mutations is evaluated for various states. **(B)** A schematic illustration on the calculation of ΔΔHB, with the mathematical definition shown in the rectangle frame. **(C)** Identification of side-chain mutants that favor the IF/OF states in XylE systems. The regions that shows strongly IF (ΔΔHB_OF→IF_ > 0.7 for both systems) and OF preference (ΔΔHB_OF→IF_ < -0.7 for both systems) are colored blue and pink, respectively. The mutants mentioned in text are highlighted in red. **(D)** Single mutations residing in blue and pink area (as well as Lys305) in (**C**) are presented in the structural view.

We designed a novel method of H-bond network modeling, named as the Interaction Regulation with Bayesian Networks (IRBN), to infer the causal relationship between all interacting residue pairs and to search for factors responsible for the evolutionary divergence using the Bayesian network, which is widely used for causality inference in omics data analysis[22]. Instead of abstracting residues as nodes, inter-residue H-bonds were symbolized as basic units to construct network models, since they can bridge network intervention and pairwise interaction energies, the latter of which jointly correlate with free energy (see Modeling of hydrogen bond networks in Methods section for details). To reduce the network complexity, H-bonds formed by identical moieties of side-chain(s) (SC) and/or backbone(s) (BB) are treated as a whole (Fig 3A; also see S7 Fig for comprehensive illustration of network learning and inference). Intuitively, the aggregate number of H-bonds in a network (defined as HB) negatively correlates with free energy, and therefore the change of HB (ΔHB) could be used to assess perturbation in free energy (ΔG) for a specific conformational state induced by certain mutations. To decipher the mutational effect on the free energy change during the transition between two conformation states (ΔΔG), we extended the concept to ΔΔHB by mimicking a thermodynamic cycle (Fig 3B).

To clarify distinct conformational preferences of XylE systems, we first constructed the network models for aligned IF, TS and OF conformations, and then disabled specific H-bonds of a polar/charged side-chain (called mutation here) in the models to calculate its ΔΔHB of OF→IF transition. Positive ΔΔHB_OF→IF_ signifies that the mutation introduces an additional bias to favor the inward-facing state (in respect of HB), relative to the outward-facing state. Fig 3C shows the ΔΔHB_OF→IF_ values of the tested mutations in XylE systems. The diagonal distribution demonstrated that the network interventions generally led to equivalent outcomes in XylE_H and XylE_noH systems. The mutations that gave rise to substantial positive or negative ΔΔHB_OF→IF_ values in both XylE systems were categorized as IF-favoring and OF-favoring respectively (Fig 3D). Mutation on either Asp27 or Arg133 favored inward-facing conformation, supporting our hypothesis that proton-coupled conformational preference originates from the modulation on Asp27-Arg133 bonding strength.

Mutations that weakened or destroyed the Asp27-Arg133 interaction, i.e. D27N and D27L, were tested by semi-quantitative PEGylation assays (see PEGylation assay in Methods section; S8 Fig). As shown in S1 Text, results of PEGylation for certain mutation plus L65C or V412C should be interpreted by comparison with L65C or V412C, respectively. Thus, we can conclude that the population of outward-facing states dramatically declined in D27N and completely diminished in D27L, comparing to WT XylE (S8C Fig). The results support that Asp27 protonation modulates conformational preference in XylE through adjusting the local H-bond network.

Other residues whose mutations showed significant conformational preference were mainly involved in inter-domain interactions, including residues located at the NTD-CTD interface and conserved cytoplasmic motifs in SP family (Fig 3D). PEGylation assays showed that notable changes could only be detected in the mutants with markedly perturbed salt bridges, such as D337L, D27L and K305M, possibly because salt bridges contribute more to free energy than regular H-bonds. Similar to D27L, the D337L mutant strongly prefers inward-facing state (S8C Fig). Despite that mutation on Lys305 (K305M) was expected to favor outward-facing conformations, its impact is weaker than D27L and D337L, considering the smaller magnitude of its ΔΔHB_OF→IF_ value (less than cutoff, see Fig 3C and S8C Fig). Since Lys305 is the only salt-bridge forming residue predicted to favor outward-facing state, we constructed double mutants combining K305M and mutations in OF-favoring region of Fig 3C including Y298F and Y179F. Consistent with model prediction, in comparison to the WT XylE, the inward-facing conformations almost disappear in the tested double mutants (S8C Fig). We thus confirm the predictive power of the newly developed methodology. Likewise, the same analysis on GLUT1 predicted multiple potential mutations for stabilizing typical conformations, which awaits further experimental validation (S8B Fig).

### Detailed mechanisms of network regulation

**Fig 4:**
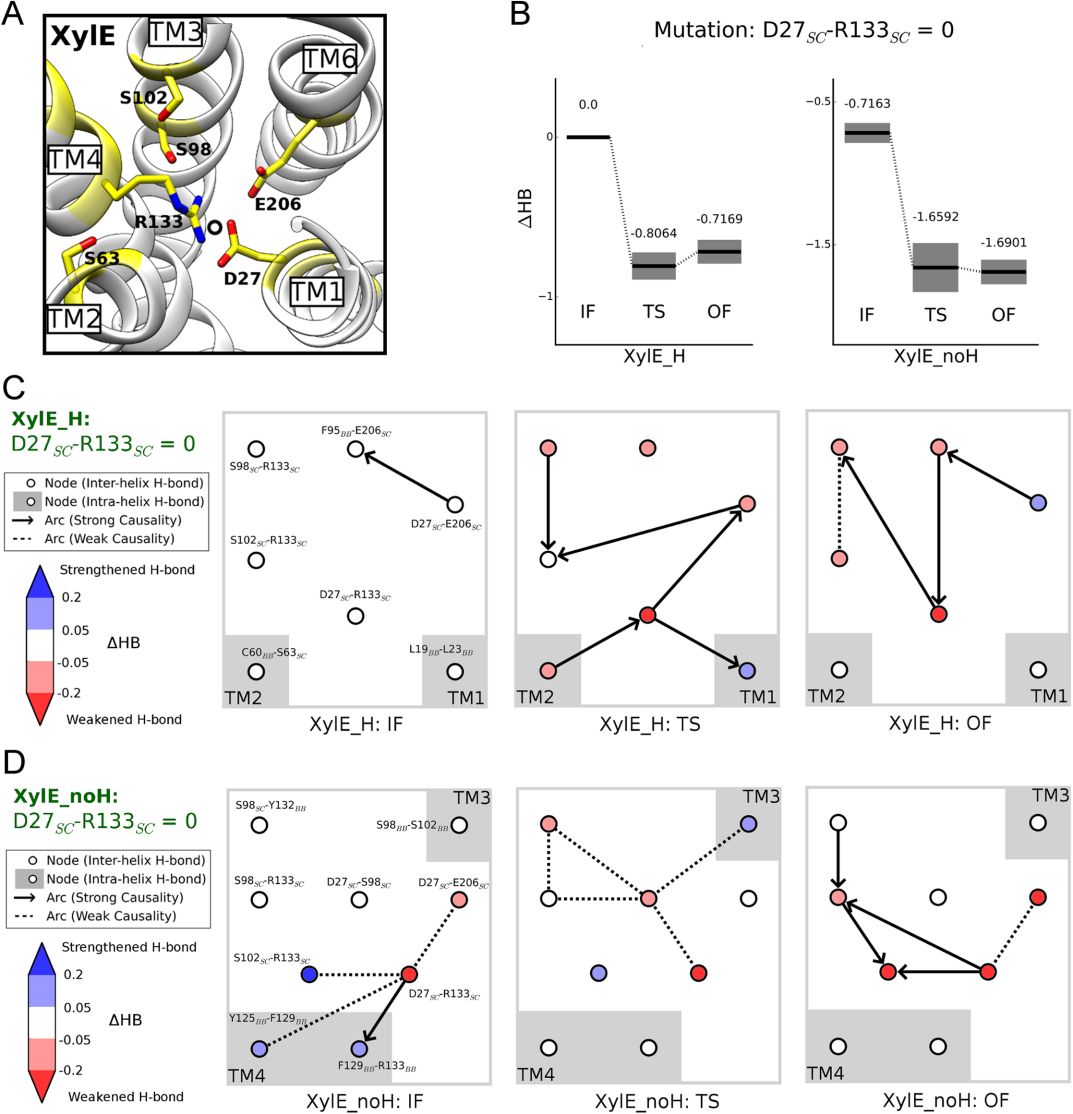
Network variations upon disrupting Asp27-Arg133 interaction in XylE systems. **(A)** Overview of local network around Asp27 inside NTD. The residues affected by forcing Asp27_SC_-Arg133_SC_ H-bonds to 0 in either XylE_H or XylE_noH are colored yellow. **(B)** Overall HB changes in IF, TS and OF states for XylE_H and XylE_noH, with error bars shown as gray shadow. **(C-D)** Details in the variations of local networks in XylE systems. The nodes with greatly perturbed HB value (|ΔHB| > 0.05) are included. The colors in the nodes reflect their ΔHB (see the color bar on the left). Arrows and dashed lines denote the strong and weak causalities between nodes, respectively. Positions of TM helices are shown as gray shadow.

To explore detailed mechanisms for the mutational perturbation on HB values, we evaluated the structure of Bayesian network models by model averaging (S7C Fig) and presented the individual nodes that were intensely perturbed upon network tuning (see Modeling of hydrogen bond networks in Methods section). Considering the coincidence of Asp27-Arg133 bonding and free energy discrepancy between XylE_H and XylE_noH (see Fig 1D and Fig 2B), we disrupted the Asp27-Arg133 side-chain H-bonds in network models but retained other interactions concerning Asp27/Arg133 (e.g., Asp27-Glu206) (Fig 4A).

Following this protocol, we investigated the change in the architecture of H-bond networks upon Asp27-Arg133 H-bond dismissal in XylE systems. When forcing the number of Asp27_SC_-Arg133_SC_ H-bonds to zero for Bayesian network inference, the overall HB value of IF state is considerably less weakened than those of TS and OF states in both systems, suggesting that breaking this interaction triggers inward-facing preference (Fig 4B). Specifically, in the XylE_H system (Fig 4C), Asp27_SC_-Arg133_SC_ disruption introduces no changes in IF state, whereas numerous inter-helix H-bonds are weakened in TS and OF states. Moreover, the upheaval of network architecture in diverse states revealed drastic variation of interaction patterns. In the XylE_noH system (Fig 4D), unlike the TS and OF states, H-bond loss regarding Asp27 in IF state (i.e. Asp27_SC_-Arg133_SC_ and Asp27_SC_-Glu206_SC_) can be partially compensated by multiple connected interactions (i.e. Ser102_SC_-Arg133_SC_ and several TM4 backbone H-bonds). We speculate that the sacrifice of these compensated H-bonds upon Asp27_SC_-Arg133_SC_ fortification may be the cause of unfavorable inward-facing conformation in deprotonated XylE.

Complete removal of the H-bonding capability of Asp27 triggers more intricate network perturbations, yet generates a comparable ΔHB pattern destabilizing TS and OF states more than the IF state (S9 Fig). However, similar treatment with Asn29 side-chain in GLUT1 does not induce strong IF or OF preference (S9D Fig), possibly because of the dramatic rearrangement of local H-bond network as exemplified by the accumulation of hydrophobic residues surrounding Asn29 in GLUT1 (S9A Fig). Therefore, multi-fold evolutionary events may have occurred to modulate the local environment surrounding Asp27 in XylE and Asn29 in GLUT1 in divergent directions.

**Fig 5:**
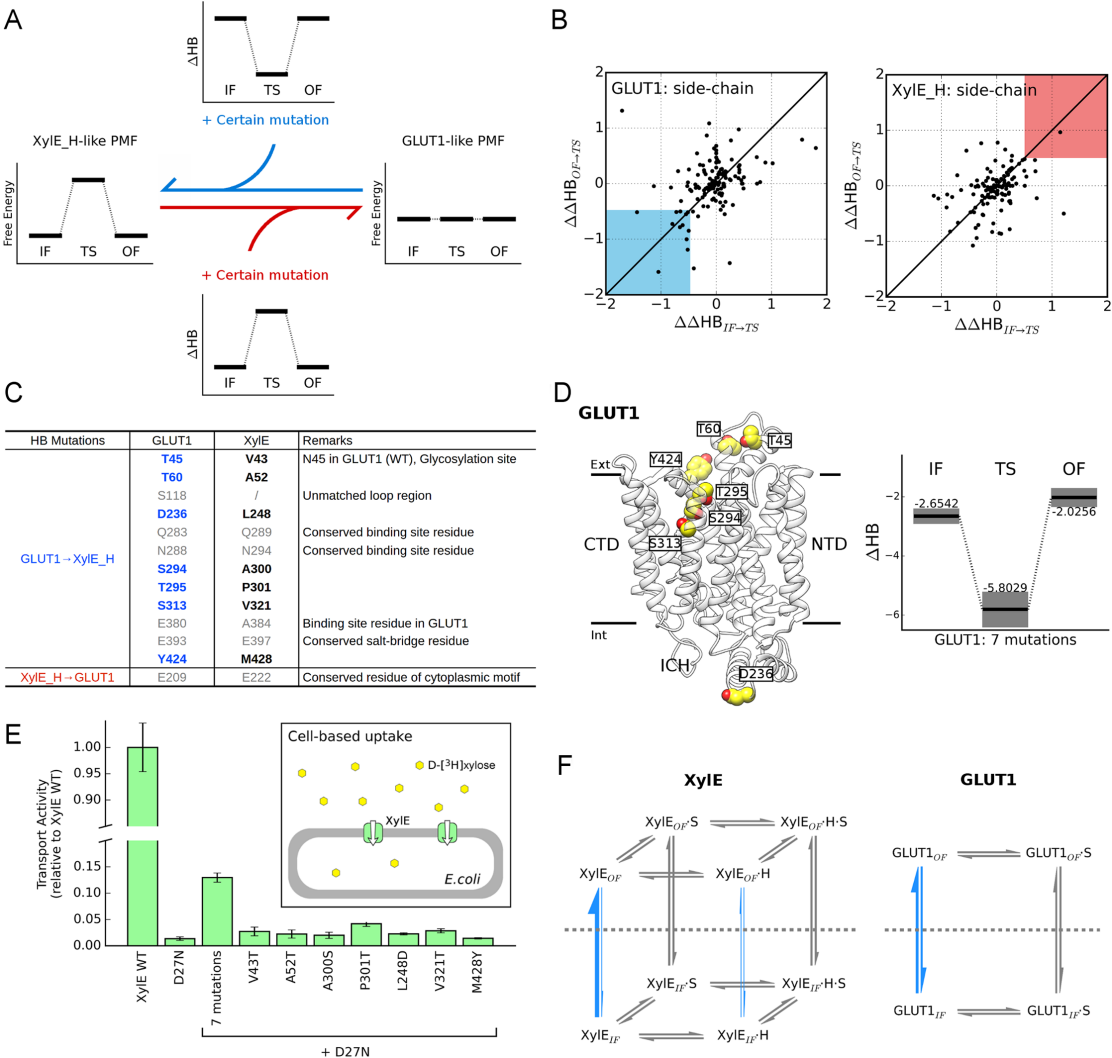
Residues responsible for thermodynamic difference between XylE_H and GLUT1. **(A)** Schematic diagram of identifying the key residues for free-energy discrepancy. The candidate residues are supposed to cause the free energy profile of one transporter to resemble the other upon mutation. Applying inference on H-bond networks, we could pinpoint mutants that reduce the energy barrier in XylE_H or introduce one in GLUT1 according to the changes in HB values. In such cases, the ΔHB of IF and OF conformations should be comparable, while ΔHB_TS_ should differ from ΔHB_IF_ and ΔHB_OF_ significantly. **(B)** (***Left panel***) Identification of GLUT1 mutants that imitate XylE_H by destabilizing TS (ΔΔHB_IF→TS_ < -0.5 and ΔΔHB_OF→TS_ < -0.5; highlight as blue region). (***Right panel***) Identification of XylE_H mutants that imitate GLUT1 by stabilizing TS (ΔΔHB_IF→TS_ > 0.5 and ΔΔHB_OF→TS_ > 0.5; highlight as red region). **(C)** Table of candidates predicted for the GLUT1→XylE_H conversion and vice versa. The mutants in gray letters are conserved, unaligned for SP family members, or critical in sugar coordination. **(D)** (***Left panel***) Structural view of GLUT1 residues identified in the left panel of (**B**). (***Right panel***) ΔHB illustration of GLUT1 possessing all 7 mutations that mimics T45V, T60A, D236L, S294A, T295P, S313V, and Y424M, as listed in (**C**). Backbone H-bonds of residue 295 were also modulated to fit T295P mutation. Error bars were shown as gray shadow. **(E)** Transport activities of XylE mutants. The effect of negative control was already subtracted (see Cell-based uptake assay in Methods section for details). Except for XylE WT, all other variants were based on D27N mutation. ‘7 mutations’ labeling indicates the combination of V43T, A52T, A300S, P301T, L248D, V321T, and M428Y. **(F)** Transport cycles of uniporter GLUT1 and proton symporter XylE. The reactions investigated in this work are highlighted by blue arrows with line widths indicating reaction rates. H and S represent proton and substrate, respectively. The horizontal gray dashed lines separate OF and IF states.

As shown in the free energy landscapes, the most crucial discrepancy between XylE and GLUT1 is the presence/absence of energy barrier at transition state. To elucidate its molecular basis, we sought for side-chain mutations in XylE_H and GLUT1 systems that would alter the energetics towards each other based on Bayesian network inference (Fig 5A). Considering the potential negative correlation between free energy and total amount of H-bonds (HB), a GLUT1 mutation that devastates H-bonds at transition state more than IF/OF states is likely to convert its free energy profile towards XylE_H pattern, and vice versa. Possible determinants for evolutionary divergence can be predicted from the values of ΔΔHB_IF→TS_ and ΔΔHB_OF→TS_, thusly.

Since it was impractical to consider all possible combinations, we only focused on screening single side-chain mutations of GLUT1 and XylE. The mutations that would generate a barrier in GLUT1 (located in the blue region of Fig 5B) and vice versa in XylE_H (located in the red region of Fig 5B), were itemized in Fig 5C. After disposal of unaligned, conserved and binding-site residues in both transporters, a total of 7 residues were identified in GLUT1 to account for its thermodynamic divergence from XylE. These residues are H-bonding donors/acceptors in GLUT1 but are replaced by nonpolar residues with comparable volume in XylE (Fig 5C). To verify their joint performance, we mimicked a combination of 7 point mutations to replace the XylE residues with the corresponding ones in GLUT1 (T45V, T60A, D236L, S294A, T295P, S313V and Y424M) in the Bayesian network models of GLUT1 and re-evaluated the HB loss in various states (Fig 5D). Just as expected, the HB value of the mutant decreases dramatically at TS state (by > 3), thus supposedly converting the flat energy landscape of GLUT1 into a XylE_H-like fashion and prohibiting rapid IF↔OF transition.

Unfortunately, we could not obtain well-behaved protein of these GLUT1 mutants required for biochemistry characterization. As a compensation, we tested the effect of these residues in the reverse direction, trying to convert XylE into a uniporter. Besides the 7 candidate residues, D27N was also introduced into XylE, and the mutants were evaluated by *in vivo* transport assays (Fig 5E). The comparable transport activity between D27N variant with negative control indicates that D27N mutation alone is insufficient for the symporter-to-uniporter conversion (see S11A Fig and S1 Text). Similarly, incorporating a single mutation (V43T, A52T, A300S, P301T, L248D, V321T, or M428Y) with D27N presented little to no activity increase. However, the combination of all mutations led to a significantly higher transport efficiency and thus indeed converted a symporter to a functional uniporter. *In vitro* counterflow assays also confirm the functionality of this 7-mutation variant comparing to uniporters GLUT1 and GLUT3 (S11B Fig). Notably, the identified residues are not confined to a sub-region of the structure (Fig 5D), implying that elaborate adjustment of interactions rather than simply altering protonation-site residues may be essential for driving uniporters and symporters to divergent directions.

## Discussion

We hereby present multiplex analyses for the sugar porter family members XylE and GLUT1 using MD simulations and a novel approach based on Bayesian networks for residue interaction analysis. The free energy calculations on the 3 systems discriminate XylE from GLUT1, and highlight the protonation state of Asp27 as the molecular basis for conformational preference alteration in XylE. Inspection of IF↔OF transitions reveals numerous details that are consistent with structural and biochemical studies, supporting the reliability of aMD+SMwST path-finding scheme.

The newly developed modeling framework IRBN plays a pivotal role in mechanism illustration. For instance, from the network models, we can predict that H-bond loss following the disruption of Asp27-Arg133 interaction in the inward-facing state of XylE_noH will be compensated by three contiguous interactions (Fig 4C). By artificially manipulating residue interactions, IRBN helped disclose molecular determinants for the uniporter/symporter divergence, hence implying that complex variations instead of mere Asp↔Asn substitution are required for the functional interconversion. As for GLUT1, we could also explain the mechanism of some disease-related mutations. As shown in S10A–S10F Fig, these mutations unanimously perturb the energetic balance among states by disrupting local H-bond network, and consequently impair fast turnover required for facilitators. Moreover, our approach of combining MD simulations and IRBN modeling could be extended to the fields of protein engineering and drug development, and could facilitate rational design as well as allosteric regulation studies for pharmaceutically important targets.

Under the guidance of computer simulation and statistical modeling, we successfully transformed a symporter (XylE) into a uniporter by neutralizing protonation site residue and reducing the energy barrier. In contrast, converting GLUT1 to a proton symporter would presumably need more intricate changes to meet following requirements: (1) ability to load and unload proton, (2) no proton leak in the absence of substrate, and (3) no substrate leak without change in the protonation state. Here, we provided several necessary tactics for the uniporter-to-symporter design, which were generalized from our calculations on thermodynamics: (1) possess a titratable residue as protonation site, (2) create an energy barrier in *apo* state, and (3) destabilize inward-facing state in deprotonated state. Notwithstanding these advances, it is noteworthy that relative free energies between IF and OF states in XylE_H and GLUT1 systems slightly disagree with experimental observations[23] for two possible reasons: (1) the applied force field did not consider polarizable dipole-dipole interactions that were supposed to fasten the extracellular gate[7], and (2) boost energies in aMD simulations partially destroyed the integrity of ICH domain in the outward-facing conformations, which should be intact as exemplified by outward-facing GLUT3 structures (PDB ID: 4ZW9, 4ZWB, and 4ZWC)[6].

In summary, we thoroughly investigated crucial reactions of the transport cycles emphasized in Fig 5F. Comparison of three *apo* systems provides detailed understanding of transporter mechanisms. Since sugar porters may recognize both α- and β-anomers as substrates, the uncharacterized processes in Fig 5F still await extensive computational and biophysical research.

## Materials and methods

### Simulation system setup and pre-equilibration

Using the plugins in VMD[24], we established three systems, i.e. Asp27 protonated XylE (XylE_H), Asp27 deprotonated XylE (XylE_noH) and GLUT1. The structure of inward-open GLUT1 mutant (N45T & E329Q, PDB ID: 4PYP) were preprocessed for MD simulations with 3 modifications: (1) Gln329 was mutated back to Glu as in WT GLUT1, (2) the detergent β-NG whose head group occupies the binding pocket was removed, and (3) the missing ICH5 (residue 459 to 468) was constructed as α-helix referring to template structures of the outward-facing XylE (PDB ID: 4GBY) and GLUT3 (PDB ID: 4ZWC and 4ZW9) using Modeller 9v12[25]. For XylE, we selected the inward-occluded structure (PDB ID: 4JA3), and modeled all missing loops using the outward-occluded conformation (PDB ID: 4GBY) as the template. PROPKA 3.1[26,27] was used to determine the protonation states of titratable residues other than Asp27 in XylE at pH 7.0. In specific, Glu206 was neutralized in both XylE systems. XylE was inserted into a palmitoyl-oleoyl-phosphatidyl-ethanolamine (POPE) bilayer, given the fact that the majority of *E*. *coli*. lipids (~75%) belong to the PE class[28],. To mimic the physiological condition for GLUT1, a palmitoyl-oleoyl-phosphatidyl-choline (POPC) membrane was adopted since it has been reported to restore transport activity of purified GLUT1[29]. After solvation and neutralization in 150 mM NaCl, the total number of atoms reached ~86,000 for each system.

We generated input files and performed simulations using MD simulation suites AMBER12[30] and AMBER14[31]. The transporters were parameterized by ff12SB force field, and were surrounded by LIPID11 phospholipids[32] and TIP3P water molecules[33]. With the protein and ligand fixed, the systems first underwent a 5000-step minimization. Then, a 1-ns melting of lipid tails was simulated in an NVT ensemble at 310 K with the rest of the system constrained with a large force constant k = 100 kcal/mol/Å^2^. Afterwards, one heating procedure (k = 10 kcal/mol/Å^2^ for protein) was carried out from 0 K to 310 K under constant volume condition for 1 ns. Next, the value of k was set to 1 kcal/mol/Å^2^ for another 1-ns pre-equilibration. To further naturalize the lipid bilayer, two 5-ns runs in the NPγT ensemble (1 atm of pressure) were performed with k = 0.1 kcal/mol/Å^2^ on Cα atoms and no constraint at all, sequentially. We deliberately selected surface tension γ for bilayers (γ = 17 dyn/cm for POPC and γ = 26 dyn/cm for POPE membrane) suggested by the reported tests of LIPID11 force field[32]. Under periodic boundary conditions (PBC), all pre-equilibrations were performed with the time step of 1 fs and the van der Waals cutoff of 10 Å, using the Particle Mesh Ewald (PME) method to estimate electrostatics[34].

### Production simulations

Initially for each system, we performed an NPγT cMD simulation for 100 ns (Sim #1, #2 and #3) using the time step of 2 fs and with the SHAKE algorithm[35] applied. Tiny fluctuations of the system volume indicated that membrane and solvent molecules were well equilibrated, and therefore we fixed volume and used the GPU implementation of PMEMD[36] for the subsequent simulations. It is usually unrealistic to sample the large-scale conformational change of transporters by cMD simulations, because of the long autocorrelation time ranging from micro- to milliseconds or even longer. Therefore, we conducted aMD simulations (Sim #4, #5 and #6) to sample the conformational transition, based on a reported study of GPCR[37]. Preserving the shape of energy surface, aMD adds a boost potential ΔV(r) to adjust total potential and/or dihedral potential[14]:

$$V{\left(r\right)}^{*}=V(r)+\Delta V(r),$$

$$\Delta V(r)=\frac{{\left(E_{p}-V(r)\right)}^{2}}{\alpha_{p}+E_{p}-V(r)}+\frac{{\left(E_{d}-V_{d}(r)\right)}^{2}}{\alpha_{d}+E_{d}-V_{d}(r)}.$$

Here E_p_ and E_d_ denote the reference values for the total and dihedral potentials respectively, while V(r) and V_d_(r) denote the total and dihedral potentials calculated for the current state of the system. The boost energy that is tuned by the parameters α_p_ and α_d_ is applied only in the situation of V < E. We set E_p_ and E_d_ as the average potentials of the second halves of the 100-ns cMD trajectories, and expressed the parameters via:

$$E_{d}=V_{d}\cdot \left(1+\lambda_{d}\right),$$

$$\alpha_{d}=\frac{\lambda_{d}}{5}\cdot V_{d},$$

$$E_{p}=V+\lambda_{p}\cdot N_{atom},$$

$$\alpha_{p}=\lambda_{p}\cdot N_{atom}.$$

After testing some combinations of parameters, we found that enhanced sampling could be visualized within 100 ns for λ_d_ = 0.3 and λ_p_ = 0.2, without perturbing the system stability (< 5% loss of helical contents). Subsequently, we extended the aMD simulations to 500 ns for all systems. Flattening energy barriers on the path, aMD allowed the protein structure to evolve faster and presented an overview of the conformational transitions.

### Path finding and refinement

For MFS transporters, identification of the pathway for the IF↔OF conformational transition typically requires an *a priori* reaction path that in principle should not deviate much from the minimum free energy path (MFEP), since otherwise even extensive iterative optimization can hardly guarantee the path convergence, particularly when an energy barrier is present between the *a priori* path and MFEP. In this work, the *a priori* paths were collected from the unbiased aMD trajectories, and therefore should be close to the MFEP in principle. This protocol simplifies the path selection process, which traditionally requires the tedious evaluation on enormous candidate trajectories produced by permuting the applying sequence of all relevant artificial collective variables. The *a priori* paths were then refined iteratively using the SMwST method until convergence.

We have utilized the GPU code of AMBER12 to run the SMwST[38] simulations in order to relax and optimize paths (Sim #7, #8 and #9). In total, 51 discretized conformations were selected for each system to compose an *a priori* pathway, including 2 end-point structures. The images were picked from previous cMD and aMD trajectories. Unlike the aMD trajectory of GLUT1, XylE_H and XylE_noH systems sampled either IF or OF conformations with slight overlapping. Hence, we combined the trajectories of Sim #4 and #5 to construct the IF↔OF transitions of XylE, and unified protonation state for each pathway. To obtain stable end points of the *a priori* path, IF or OF snapshots with the largest Intracellular/Extracellular Gate distances were chosen and were then equilibrated for 5 ns following a 10,000-step minimization. The initial path connecting 2 end points should meet three criteria described as follows:

1. Using Sim #4 & #5 for XylE and Sim #6 for GLUT1, we conducted a principal component analysis for the Cartesian coordinates of Cα atoms in the TM domain. 50 tandem intervals with equal length were first set up between the 2 end points along the first principal component, and the intermediates were then selected around the interval junctures within a radius equal to 20% of the interval length.
2. Cα-RMSDs of the TM domain between the i^th^ image and its neighbors should be less than 1.4 Å.
3. Among all possible paths that satisfied criteria (i) and (ii), the one with images having the lowest total aMD-boost-energies was taken as the initial path.

To ensure continuity in high-dimensional space, we used Cartesian coordinates of the Cα atoms as the collective variables for SMwST calculation. The iterative method mainly follows a 4-step procedure as published[39,40]:

1. For each image, following a 1000-step minimization, 100 2-ps equilibrium simulations with random seeds were launched.
2. The final structures from the swarms of trajectories were then superimposed to calculate the average drift in the collective-variable space.
3. Images on path may become unevenly distributed after drifting. Therefore, the drifted path has to be adjusted by creating new image structures in an evenly separated manner. Coordinates of the new images z^i*^ were estimated by a linear interpolation between those of two neighboring old images z^k-1^ and z^k^:
  $$z^{i^{*}}=z^{k-1}\cdot \frac{L(k)-S(i)}{L(k)-L(k-1)}+z^{k}\cdot \frac{S(i)-L(k-1)}{L(k)-L(k-1)},$$
  where S(i) denotes the expected image separation for the i^th^ image in the drifted path, while L(k) denotes the image separation for the k^th^ image in the old path.
4. With a strong force constant (40 kcal/mol/Å^2^), the image structure with coordinate z^i^ of the i^th^ image was guided to its expected coordinate z^i*^ in the collective-variable space by running a 50-ps constrained equilibrium simulation.

The above procedure was repeated for 60 iterations to iteratively refine the path. Distance between two paths could be evaluated by the summation of pairwise RMSDs between corresponding images. In the convergence tests, the path of the current iteration was compared with the initial path (named as std 1) and that of 4 iterations before (named as std 2). The path-finding algorithm is thought to reach convergence if both distances become nearly constant, independent of the iteration index. By comparison with the convergence scenario in [40], the average drifts in RMS space not only reach plateaus more rapidly in our cases, but also hold lower values (<1.5Å vs. >3Å). The observation indicates that initial paths found by aMD are sufficiently close to MFEP and are likely to outperform those sampled from TMD used in [40]. The final SMwST paths were used to initiate follow-up calculations.

### Free energy calculations

We introduced 2D reaction coordinates for PMF calculations based on *a priori* knowledge. As NTD and CTD undergo nearly rigid-body rotation in line with the alternating access mechanism, we simplified the metric of IF↔OF transitions to Extracellular and Intracellular Gate distances, both of which quantify the gate opening using separation between the centers of mass (COM) of two Cα groups. Specifically, residues of the two groups describing XylE Extracellular Gate, XylE Intracellular Gate, GLUT1 Extracellular Gate and GLUT1 Intracellular Gate are listed below: (1) XylE Extracellular Gate: residue group {28–34, 58–63, 178–183} vs. residue group {295–301, 315–320, 423–428}, (2) XylE Intracellular Gate: residue group {75–80, 149–154, 160–166} vs. residue group {332–337, 391–397, 404–410}, (3) GLUT1 Extracellular Gate: residue group {30–36, 66–71, 171–176} vs. residue group {289–295, 307–312, 419–424}, and (4) GLUT1 Intracellular Gate: residue group {83–88, 141–147, 153–159} vs. residue group {324–329, 387–393, 400–406}, respectively.

The free energy calculations were performed in the BEUS scheme[41]. The method has a feature of swapping adjacent windows/replicas with moderate acceptance ratio to improve sampling continuity. Ideally, images of a convergent SMwST path can serve as the window centers for BEUS simulations. For complex systems with high dimensionality, however, hundreds of swarmed trajectories in the SMwST calculations are far from sufficiency to yield a drift that depicts the real gradient. Therefore, SMwST images could stray from MFEP. To overcome this problem, we performed a 5-ns equilibrium simulation for each image to generate an ensemble of structures around the SMwST path, and then used a curve reconstruction algorithm[42] to regenerate the path in the 2D space of gate distances (S2A Fig).

The 5-ns equilibrium simulations (Sim #10, #11 and #12) can not only facilitate path reconstruction, but also produce sufficient conformations to shed light on the positions of putative energy barriers and basins. To estimate the PMF profile of an IF↔OF transition, the reconstructed path was divided into 30 windows, in each of which the simulated structures were restrained in the space of gate distances by a biasing potential $U(\zeta )=\frac{k}{2}\cdot {\left(\zeta -\zeta (s)\right)}^{2}$, where ζ is the space coordinate, ζ(s) represents the center of window and k is the force constant. The window exchange was initiated every 20 ps following a deterministic odd-even scheme[43]. For each window exchange, two neighboring windows swapped their centers following an acceptance probability as described below:

$$P_{ij}=\min\left\{1,\exp(-\Delta )\right\},$$

$$\Delta =\frac{1}{k_{B}T}\left\{\left[U_{i}(X_{j})+U_{j}(X_{i})\right]-\left[U_{i}(X_{i})+U_{j}(X_{j})\right]\right\},$$

where P_ij_ is the acceptance probability for the swapping between window i and j, k_B_ is the Boltzmann constant, T is the absolute temperature, U is the potential energy and X is the Cartesian coordinate. Test simulations were conducted on the conformations collected from trajectories of Sim #10, #11 and #12 to optimize the window centers and corresponding force constants, so as to guarantee moderate exchange rates of 20–40% between all neighboring windows. Ultimately, we conducted 30 replicas × 20 ns/replica = 600 ns of BEUS simulations for each path. Due to limited computing resources, we produced the final PMF profile using the last 16 ns trajectories in all BEUS windows, which included 24,000 structures (30 windows × 800 snapshots/window) in total. Bayesian block bootstrapping scheme in combination with the weighted histogram analysis method (WHAM) was used for the estimation of PMF and error bars[44,45]. For a BEUS replica, we measured its autocorrelation times in dimensions of the reaction coordinates and window index. The maximal autocorrelation time τ is less than 2 ns. We thus divided the BEUS time series within one window into 4-ns non-overlapping blocks. Notably, sizes of blocks were at least twice of τ, thus avoiding inter-block correlation. The block sampling technique resolves the violation of independent and identical distribution case by standard bootstrapping, and provides better uncertainty estimations as discussed in previous literatures[16,17,41].

### Window alignment

The BEUS windows of XylE_H, XylE_noH and GLUT1 need to be properly aligned before pathway comparison. Following superimposition, the structures in each BEUS window were averaged to generate a representative conformation for the window. Therefore, a total of 30 representative structures were generated for each path. Distances between representative structures were evaluated by RMSD. To estimate RMSD between XylE and GLUT1, the structures should be superimposed on their matching residues in the TM domain. In specific, residue {12–35, 66–84, 91–111, 122–174, 186–207, 272–297, 307–357, 367–401, 411–428, 434–451} in GLUT1 matches the residue {10–33, 59–77, 84–104, 130–182, 200–221, 279–304, 316–366, 372–398, 408–425, 446–463} in XylE.

Ahead of multi-path alignment, we first performed pairwise alignment by calculating pairwise RMSDs between the representative conformations of the two paths (S3A Fig). Here, we recorded the results in a 30 by 30 matrix denoted as A, where A[i, j] is the RMSD between window i of path 1 and window j of path 2. We then generated legitimate routes between the end points A[0, 0] and A[29, 29], requiring that as long as A[i, j] is on a route, either A[i+1, j] (i∈{0, 1, …, 28}, (j∈{0, 1, …, 29}) or A[i, j+1] (i∈{0, 1, …, 29}, (j∈{0, 1, …, 28}) should stay on the route. By this way, any route including (i, j) would have the form of {(0, 0), …, (i, j), (i+1, j), …, (29, 29)} or {(0, 0), …, (i, j), (i, j+1), …, (29, 29)}. We assume that the optimal route should have the minimal summation of RMSDs ($\sum_{(i,j)\in route}A[i,j]$). Note that this definition does not infer a one-to-one mapping between representative structures.

In the multi-path alignment, the windows from 3 paths were regarded as matched, only when every pair of them was connected by the above route finding algorithm (S3B Fig). Among the one-to-many mapping of the 3-window sets representing the same conformation, the one with the lowest summation of pairwise RMSDs was chosen to guarantee the uniqueness of state matching.

### Analysis techniques

To evaluate the conformational changes, we conducted principal component analysis for GLUT1, XylE_H and XylE_noH systems using structural snapshots in the aligned BEUS windows (S4A Fig). Ahead of principal component analysis, the Cα atoms of residue {12–35, 66–84, 91–111, 122–174, 186–207} in GLUT1 and residue {10–33, 59–77, 84–104, 130–182, 200–221} in XylE systems were used to align the structures upon the NTDs. The same TM atom set as adopted in the preceding section (“Window alignment”) was used here to estimate the principal components.

To estimate the kinking angle of TM7b, we first split this helix into two 7-residue short helices, i.e. residue 287–293 and 294–300 in GLUT1, residue 285–291 and 292–298 in GLUT3, as well as residue 293–299 and 300–306 in XylE. We then constructed an ideal α-helix using (Ala)_7_ and superimposed this ideal helix to the two halves of TM7b respectively. The axes of the two superposed ideal helices could be used to estimate the kinking angle (S6 Fig).

Pore radii were calculated using the program HOLE2[46,47] with default parameters. H-bonds were evaluated using the “Hydrogen bonds” plugin of VMD, with the angle and distance cutoffs chosen as 45° and 3.0 Å respectively.

### Modeling the hydrogen bond networks

The networks of residue interactions imply crucial details in the IF↔OF transitions. Among all kinds of interactions, we studied H-bonds explicitly, considering the easiness of mutation and experimental verification. We developed a modeling procedure named IRBN, which constructs Bayesian network models for the aligned BEUS windows.

Any system can be regarded as a set of interdependent component, which are described by random variables X_1_, X_2_, …, X_n_. Using Bayesian networks, causality relationship between the components can be inferred and the joint probability distribution of all random variables can be estimated, given sufficient observation data.

Ideally, one may attempt to fully describe a high-dimensional problem using the joint probability distribution P(X_1_, X_2_, …, X_n_) obtained from the observations (i.e. data set). However, this is impractical to handle computationally for large n because the number of parameters increases exponentially. Taking each variable as a node, Bayesian networks factorize the joint distribution and assign inter-node arcs according to conditional dependencies, thereby reducing the complexity and revealing causality between nodes. The obtained Bayesian network models provide mass knowledge on information propagation through nodes and supports probabilistic reasoning at the given evidence.

Here, we only investigated the H-bond networks for the IF, TS and OF states. In total, 3 window (representing IF, TS, OF states) × 3 path = 9 windows, each of which has a sample size of 1000, were studied. The Bayesian network models were trained using BEUS trajectories, where the conformations were saved every 20 ps for 20 ns. Note that 20 ps is sufficiently longer than the autocorrelation time of H-bond rearrangement. We determined the H-bonds using simple geometric criteria with the angle and distance cutoffs chosen as 45° and 3.0 Å respectively. For a protein structure, each residue was decomposed into side-chains (SC) and backbones (BB), either of which could act as a moiety to form H-bond interaction. Logically, we articulate a random variable (node) as the number of H-bond between two such moieties. For example, the number of H-bonds formed between the backbone of Asn29 and the side chain of Arg126 in GLUT1 is defined as a discrete random variable called Asn29_BB_-Arg126_SC_, which has a value range of VAL(Asn29_BB_-Arg126_SC_) = {0, 1, 2}. The H-bonds that occur in less than 10% of the structural snapshots were considered trivial for a particular window, thus were not involved in the network. Also, H-bonds involving certain loop regions that have RMSFs larger than 2.0 Å (XylE: residue 106–123, 430–442, 465–480, GLUT1: residue 454–469) as shown in S5A and S5B Fig, were not taken into account, because the 20-ns BEUS simulations cannot guarantee complete sampling in the conformation space of these loops. Following the above requirement, each network contains 400–500 nodes. To speed up structure learning of the networks, it is reasonable to assume that two nodes are disconnected if their moieties do not contact. By considering physical interactions, an arc blacklist was constructed, and only node pairs outside the blacklist could be connected by arcs (called candidate arcs) in the following Bayesian network learning. Here, physical contact is counted either if on average > 1 pairs of heavy atom proximate within the cutoff of 4.0 Å, or if nontrivial H-bonds are found. As a result, we implicitly included the van der Waals interactions into network modeling. The regime of IRBN is thoroughly illustrated in S7 Fig.

We used the bnlearn[48] and gRbase[49] packages in R for the learning and inference of Bayesian network. Model construction and parameter estimation followed a two-step process: (1) structure learning to generate the structure of the directed acyclic graph (DAG), and (2) parameter learning to determine the local probability distributions. For discrete Bayesian networks, we performed structure learning using a hybrid algorithm named general 2-Phase Restricted Maximization (RSMAX2), which is a combination of constraint- and score-based algorithms. We chose the Semi-Interleaved HITON-PC for the restrict step (phase 1), which is optimal for high-dimensional data sets[50]. In detail, the conditional independences of nodes were evaluated by the mutual information tests using semi-parametric χ^2^ distribution[51] (α = 0.01 as the type I error threshold). After learning the skeleton of the DAG by Semi-Interleaved HITON-PC, the structure underwent fine-tuning by a score-based tabu search (length of tabu list = 50). The score of the Bayesian Dirichlet equivalent uniform (BDe) posterior density (imaginary sample size = 5) was maximized in tabu search in the structural tuning process[52]. Once the highest score structure was learned from data, the step of parameter learning was executed by the commonly used Bayesian estimation with imaginary sample size of 5. In rare cases, the nodes representing nontrivial H-bonding that comprises of constant number of H-bonds in all snapshots were added back to the network manually after model learning. It is worth mentioning that the parameters and algorithms described above were chosen based on 5-fold cross-validation results in one dataset (the window of IF state in GLUT1). Setting log-likelihood loss (also known as negative entropy) as our loss function, we evaluated the combinations of following items: (1) cutoff for physical contacts (0.5, 1, or 2 pairs of contiguous heavy atoms), (2) constraint-based algorithms named Max-Min Parents and Children (MMPC), or Semi-Interleaved HITON-PC for phase 1, (3) score-based algorithms denoted as hill-climbing or tabu search for phase 2, and (4) the significance level of dependency test (α = 0.01, 0.02, or 0.05). According to the testing results, the loss function is insensitive to the choice of parameters/algorithms. We thus selected aforesaid combination that has relatively small loss and moderate number of arcs.

The features of a Bayesian network model can be estimated by resampling techniques. Here, we performed 10 times of 5-fold cross-validation on each dataset, thereby generating a total of 50 Bayesian network models (each constructed from 80% of the dataset) for feature estimation and model averaging (S7C Fig). Under the assumption that net change of H-bonds correlates with the free energy change ΔG, we defined a network feature as HB to depict the expectation of the total number of H-bonds:

$$HB=E\left(\sum_{i}X_{i}\right)=\sum_{i}E(X_{i}).$$

Clearly, HB can be estimated as the summation of E(X_i_), which can be computed from the marginal distribution of random variable X_i_ at each node. To measure the HB change, i.e. ΔHB of a network caused by specific mutations, one can perform Bayesian network inference at a given evidence

$$Evi=\left\{X_{j_{1}}=e_{1},X_{j_{2}}=e_{2},\ldots ,X_{j_{k}}=e_{k}\right\},j_{1},j_{1},\ldots ,j_{k}\in \left\{1,2,\ldots ,n\right\},$$

which quantitatively describes random variables affected by the mutations, and calculate conditional distribution P(X_i_ | Evi). Consequently, the expression of ΔHB is written as:

$$\Delta HB=HB^{Mut}-HB^{Ori}=\sum_{i}\left[E(X_{i}|Evi)-E(X_{i})\right],$$

where HB^Ori^ denotes the HB of original unperturbed Bayesian network model, while HB^Mut^ denotes the HB of Bayesian network model after mutational intervention. Furthermore, we can investigate the efficacy of some mutations on the transition between two states, such as ΔΔHB_OF→IF_ = ΔHB_IF_ - ΔHB_OF_, which imitates ΔΔG of a thermodynamic cycle.

In the investigation on information propagation, we applied model averaging using 50 Bayesian network models to guarantee the robust network structures. An arc was included in the averaged DAG only if it emerged in > 90% of the 50 models. In addition, the arc was considered directed if the network connection shows evident directionality (proportion of same direction > 80%). In the graphical representation of the robust network (see Fig 4C and 4D and S9B and S9C Fig), the highly oriented arcs representing strong causality are shown in arrows, while the other arcs are represented as dashed lines.

### PEGylation assay

Methoxypolyethylene glycol maleimide 5,000 (mPEG-Mal-5K; Sigma-Aldrich), serving as a membrane-impermeable PEGylation reagent, can be covalently attached to solvent accessible thiol groups, thus change the mobility of molecules in electrophoresis. In spite of its native cysteines, WT XylE embedded in *E*. *coli* membranes shows no shifting on gel after mPEG-Mal-5K treatment, suggesting that mPEG can access none of the native thiol groups. We introduced a cysteine at Leu65 or Val412 in combination with certain mutations for inspection. The specific sites of L65 and V412 were selected based on solvent accessibility analysis of simulated conformations, as L65 or V412 is only exposed in OF or IF state, respectively. All XylE mutants were subcloned into pET15b (Novagen) with an N-terminal 6 × His tag. *E*. *coli* BL21 (DE3) cells were used as expression system and were incubated in shakers at 220 r.p.m. at 37°C. When OD_600_ of Luria-Bertani medium reached 1.5, overexpression was induced by 250 μM isopropyl β-d-thiogalactoside (IPTG) for 4 h. The cells were harvested and washed twice before resuspension by PEGylation buffer containing 25 mM HEPES-Na pH 7.5, 150 mM NaCl and 10% (v/v) glycerol. Reaction system comprised of 0.1 g/ml cells with or without sonication and 10 mM mPEG-Mal-5K in PEGylation buffer. The mixtures were gently shaken for 1 h at 20°C. Then the reaction was quenched by 20 mM dithiothreitol (DTT) followed by SDS-PAGE and western blotting analysis (S8C Fig).

### Cell-based uptake assay

XylE variants were subcloned into pET15b (Novagen) and transformed into *E*. *coli* BL21 (DE3) cells for transport activity measurements *in vivo*. *E*. *coli* cells were induced with 250 μM IPTG for 1 h, when OD_600_ of Luria-Bertani medium reached about 1.5. Ice-cold MK buffer (150 mM KCl, 5 mM MES-K pH 6.5) was used to wash the harvested cells twice and subsequent resuspension. To energize the cells, we added glycerol to a final concentration of 10 mM, and left the reaction system at room temperature (25°C) for 2 min right before sugar uptake. Cell density of the 100 μl reaction system should be adjusted to OD_600_ = 2.0. The transport reaction triggered by 0.83 μM extracellular D-[^3^H]xylose addition was allowed for 30 seconds at room temperature, and was stopped by rapid dilution in 2 ml of ice-cold MK buffer followed by filtration through 0.22-μm cellulose acetate filter (Sartorius) and 2 ml MK buffer washing. The filter membranes were taken for liquid scintillation counting. Another MFS transporter FucP, which is unable to transport D-xylose, served as negative control. All the XylE mutants were expressed on similar levels quantified by western blotting.

### Liposome preparation and counterflow assay

We mainly followed previous protocols for protein purification and liposome preparation[5,6,7] with minor modifications. N-his tagged 7-mutation variant was extracted from *E*. *coli* BL21 (DE3) cells with the addition of 20 mM D-xylose throughout the purification procedure. GLUT1 was tagged with N- terminal FLAG for better behavior on gel filtration. The liposomes were reconstructed in KPM 6.5 buffer (50 mM potassium phosphate, 2 mM MgSO_4_ pH 6.5), with 20 mg / ml E. coli polar lipids (Avanti), 1% β-OG (Anatrace), 200 μg / ml protein, and 20 mM D-xylose or D-glucose. Liposomes containing no protein served as negative control. After detergent removal, extrusion and ultracentrifugation, proteoliposomes were re-suspended with ligand-free KPM buffer right before transport assays. Counterflow assays were performed at room temperature (25°C). The external concentration of hot substrates was 0.83 μM for both D-[^3^H]xylose (12 Ci / mmol) and D-[^3^H]glucose (20 Ci / mmol). The uptake was allowed for 30 s, followed by rapid filtration through 0.22-μm filters (Millipore) and liquid scintillation counting.

## Supporting information

S1 Text Discussion on various topics. (PDF)

S1 Fig RMSD calculations on the TM domains of cMD, aMD and BEUS trajectories. **(A)** TM-domain Cα RMSD of cMD simulations (Sim #1, #2 and #3) in comparison with the initial structures. The trajectories of GLUT1, XylE_H and XylE_noH are colored red, blue and yellow respectively. The steady RMSD time-series with fairly low values (< 1.5 Å) indicate system stability and modest conformational shift for the 3 systems. **(B-D)** TM-domain Cα RMSD of aMD simulations (Sim #4, #5 and #6) in comparison with reported crystal structures. GLUT1 (**D**) transits from IF to OF state, since its RMSD with respect to the outward-facing GLUT3 (PDBID: 4ZWC) structure nearly monotonously drops with time. XylE_H (**C**) and XylE_noH (**D**) systems show strong preference for IF and OF conformations respectively, since they mainly sample in the RMSD-based spaces around either inward-open (PDB ID: 4JA4) or outward-occluded (PDB ID: 4GBY) structure of XylE. **(E)** TM-domain Cα RMSDs of all structure in each window were calculated for the estimation of mean value and standard deviation. The RMSD profiles along the transition path suggest that all 3 systems sequentially visit the IF, Occ and OF conformations. It is worth mentioning that structures in windows near the end of the path could be more widely open than the OF crystal structures (PDB IDs: 4ZWC and 4GBY). **(F)** PMF profiles with windows of highest structural similarity to known crystal structures labeled as dots.
(TIF)

S2 Fig Path-finding and free energy profiles in collective-variable space. **(A)** Unweighted contour maps in the space of gate distances, constructed from the equilibrium simulations (Sim #10, #11 or #12). The yellow lines represent the transition paths reconstructed in the space of gate distances, based on equilibrium simulations (see Free energy calculations in Methods section for details). **(B)** Convergence analysis for the SMwST simulations, using std 1 and std 2 as defined in Methods. **(C)** PMF profiles in the 2D space of gate distances. The positions of IF, TS and OF states (see S3C Fig for definition) are shown as red dots.
(TIF)

S3 Fig BEUS window alignment. **(A)** RMSD-based scoring matrix of pairwise path alignment. Average structures of each BEUS window are used to generate a discretized pathway of certain systems. After computing the RMSDs of TM-domain Cα atoms between corresponding representative structures on two paths, we could define a route with minimal summation of RMSDs as aligned. **(B)** Multi-path alignment of GLUT1, XylE_H and XylE_noH systems. Multi-path alignment is constructed by pairwise alignment results, requiring that only those window triplets bearing lowest summation of RMSD are considered as aligned (see Window alignment in Methods section for details). In total, 19 aligned window triplets were obtained and they were re-numbered. **(C)** PMF profiles of the aligned windows. Once the BEUS windows are aligned, we can define IF, TS and OF states clearly, as aligned window A3, A12 and A16 respectively, by referring to the free energy profiles.
(TIF)

S4 Fig Global conformational changes along the transition path. **(A)** Correlations between top 5 principal components (PCs) of the 3 systems. **(B)** Visualization of GLUT1 and XylE_H in distinct conformations. Noticeably, rocker-switch motion of NTD and CTD takes place for both systems (also see S1 Movie). **(C)** Conformational changes of TM helices during the extracellular (periplasmic) and intracellular (cytoplasmic) gating. Red arrows indicate the significant changes in distance between TM helices during gating. The dashed lines show the interface between NTD and CTD.
(TIF)

S5 Fig The intra-domain local conformational changes along the transition path. **(A-B)** RMSFs for the average structures of all aligned BEUS windows. The structures were superimposed upon NTD or CTD before the calculation. Large RMSF values indicate significant local conformational change along the transition path. Regions fluctuating vigorously include numerous loops as well as TM7b. **(C)** Structure alignment of NTD and CTD in IF (blue) and OF (red) states for GLUT1 and XylE_H. TM helices in the back are uncolored for clarity. The resemblance between IF and OF states supports nearly rigid-body movement.
(TIF)

S6 Fig TM7b kinking and gating. **(A)** (***Left panel***) TM7b kinking in the representative structures of IF (blue) and OF (red) states for GLUT1. Definition of the kinking angle is illustrated on the IF structure. OF state of GLUT3 crystal structure is shown in gray for comparison. (***Right panel***) TM7b kinking in the representative structures of IF (blue) and OF (red) states for XylE_H (left) and XylE_noH (right). Representative structure of the aligned window A18 is shown in gray for comparison. The yellow arrow labels the place where the kinking begins to diminish in window A18. Pro301 that is supposed to facilitate kinking is shown as yellow sticks in the IF structure. **(B)** The kinking angle of TM7b as illustrated in (**A**) along the aligned transition paths of GLUT1 (red), XylE_H (blue) and XylE_noH (yellow). The kinking angle in the OF state of GLUT3 crystal structure is shown as black dashed line for reference. **(C)** Profiles of pore radii along z-axis in the IF (blue), TS (green) and OF (red) states. The vertical dashed lines represent the minimal radius to allow substrate (D-glucose/D-xylose in chair conformation) access. In XylE systems, pore radii of window A18 (see S3C Fig for definition) are also appended (black). Regions of the Extracellular Gate (EG), Intracellular Gate (IG) and substrate binding sites are shown in gray shadow. The black arrow denotes the unique gate identified in the XylE systems.
(TIF)

S7 Fig Schematic diagram of Bayesian network modeling. **(A)** Structure and parameter learning of Bayesian network modeling. (***Left panel***) Given an aligned BEUS window, we identified the non-trivial H-bonds, which were then combined into side-chain and backbone moieties. (***Middle panel***) Non-trivial H-bonds were first symbolized as nodes. An arc blacklist was then generated that included the node pairs whose moiety never formed physical contacts in the BEUS simulations. (***Right panel***) Through training on the dataset (structures in the BEUS window), the structure of the network was established with directed arcs showing causal relationships (or dependencies), and the conditional probability for each directed arc was estimated. **(B)** Bayesian network inference based on hard evidence. (***Left panel***) The marginal distributions for each node were used to calculate the expected value of H-bond number for each node. The total amount of H-bonds in the original network, denoted as HB^Ori^, was derived by summing over nodes in the network. (***Middle panel***) With a hard evidence (e.g., Asp27_SC_-Arg133_SC_ = 0), the marginal distributions of some random variables change, which therefore describes effect of specific mutations. The conditional expected value of each node conditioned on this evidence was calculated. The total amount of H-bonds in the perturbed network, denoted as HB^Mut^, was then derived by summing over nodes. (***Right panel***) Based on the Bayesian network model constructed from the corresponding BEUS window, ΔHB = HB^Mut^–HB^Ori^ can be calculated for one state, which quantitatively evaluates the overall H-bond change. Finally, ΔΔHB is derived to evaluate the influence of specific mutations on the transition between two states (see Fig 3B for details). **(C)** (***Left panel***) Estimation of a Bayesian network (BN) feature denoted as f(BN) by cross-validation (HB in our case). Mean and standard deviation could be easily derived from the models learned from resampled data. (***Right panel***) These Bayesian network (BN) models were averaged to generate a robust Bayesian network model, requiring that an arc was included in the averaged DAG only if it emerged in > 90% of all Bayesian network models.
(TIF)

S8 Fig Side-chain mutations predicted to favor IF/OF in GLUT1 and the validation of a few XylE mutants by PEGylation assays. **(A)** Solvent accessibility of the PEGylation sites L65C and V412C at certain states. L65C and V412C can only be labeled by mPEG from periplasmic and cytoplasmic sides respectively. **(B)** Predicted GLUT1 side-chain mutations that strongly prefer IF and OF states. The residues favoring OF and IF should satisfy the individual condition of ΔΔHB_OF→IF_ < -0.7 and ΔΔHB_OF→IF_ > 0.7 respectively (see Fig 3C for details). **(C)** Western blot results of PEGylation assays. Control groups indicate that WT XylE has no available cysteines for labeling, while L65C and V412C can be modified without and with sonication step to break the cells respectively. Mutants favoring IF and OF conformations are colored blue and red respectively.
(TIF)

S9 Fig Network variations upon turning off Asp27_SC_/Asn29_SC_-related interactions. **(A)** Structural views of the local network around Asp27/Asn29 inside NTD. In each system, the residues affected by network intervention based on Bayesian network inference are colored yellow. Asp27_SC_ and Asn29_SC_ are decolorized to emphasize the removal of all relevant interactions. **(B-C)** Detailed variations of influenced local networks in XylE systems. Similar to Fig 4C and 4D, the robust Bayesian network models were generated by model averaging (see S7C Fig). **(D)** ΔHB profiles in IF, TS and OF states for the 3 systems. The error bars in gray represent the standard deviations that were calculated by resampling technique (see S7C Fig).
(TIF)

S10 Fig Disease-related mutations of GLUT1 and reactions in transport cycles. **(A-E)** ΔHB profiles of disease-related mutations. In all mutations, the polar/charged residues are substituted by hydrophobic ones, which therefore disable side-chains H-bonds. According to Bayesian network inference, all of these mutations substantially alter the energetic balance between IF and OF states, thereby presumably devastating the transport activity of GLUT1. **(F)** Summary of the variants. The listed mutations were discovered to cause ‘GLUT1 deficiency syndrome 1/2’ (DS1/2) or ‘epilepsy, idiopathic generalized 12’ (EIG12).
(TIF)

S11 Fig Transport cycles concerning cell-based and counterflow assays. **(A)** Transport cycles that could generate a net influx of radioactive substrate. (***Left panel***) Uptake of a hot substrate. (***Right panel***) Exchange a hot substrate for an internal cold one. (**B**) Counterflow activities of XylE 7-mutation variant, GLUT1 and GLUT3. The negative control has already been subtracted.
(TIF)

S1 Movie Global transition and local rearrangement of three systems. The video illustrates the morph of three transporters traversing all 19 aligned BEUS windows along the transition path. Residues around Asp27-Arg133 in XylE or Asn29-Arg126 in GLUT1 are presented in sticks in bottom panels.
(MPG)
