# Supplementary material for: Molecular determinants for the thermodynamic and functional divergence of uniporter GLUT1 and proton symporter XylE
Source: PLoS Comput Biol. 2017 Jun 15;13(6):e1005603. doi: 10.1371/journal.pcbi.1005603 (PMC5491310; doi:10.1371/journal.pcbi.1005603)
Supplement: S1 Text — (PDF) [file pcbi.1005603.s001.pdf]

# S1 Text. Discussions on various topics.

## Crystal structures do not necessarily locate at PMF minima as we calculated.

We pinpointed all the window conformations closest to certain known crystal structures in S1E Fig, and created a new panel S1F Fig. Obviously, many of them locate away from local minima of 1D PMF, especially GLUT1 (IF) 4PYP as reviewer pointed out. In general, biomolecule conformations at energy minima in crystalline and in physiological environment are not necessarily identical. Factors such as protein mutations, detergents, crystal packing and other solvent molecules could all be influential, and they are actually being used as crystallization tricks to trap proteins in desired states. In the case of GLUT1, two elements mainly contributed to the stabilization of IF conformation:

(1) Introduction of the single point mutation E329Q whose equivalent was predicted to stabilize IF conformation in GLUT4[1].

(2) The presence of detergent β-NG in the central cavity. The aliphatic tail actually prevents the closure of the two domains on the intracellular side.

To mimic physiological conditions, we made corresponding adjustments ahead of system construction as described in Methods section.

## D27 protonation efficiently inhibits IF↔OF transition of *apo* XylE.

A 5 kcal/mol barrier for XylE_H system (Fig 1D) appears to be lower than what we expect. As reported[2], PMF peak value in low dimensional space only provides lower boundary to the real barrier, due to degeneracy of dimensions in which PMF is estimated. Therefore, we cannot exclude the possibility of underestimating the energy barrier, since our BEUS calculations were conducted in 2D collective variable space. Including more dimensions would alleviate this problem, but may bring more new troubles, such as limited sampling.

In the lack of experimental kinetic data for the *apo* XylE, we can only provide a rough estimation of the reaction rate constant for protonated XylE (XylE_H), using the kinetic data of GLUT1. According to previous study[3], the rate constant for IF-to-OF transition of the barrier-less GLUT1 is 147 $s^{-1}$ at 25℃. By taking this number as the pre-exponential constant in the equation of transition state theory, the corresponding rate constant of *apo* XylE_H system with a barrier of 5 kcal/mol is $147 \times \exp(-5 / RT) \approx 0.034\ s^{-1}$ at room temperature, which is roughly equivalent to an average transition time of 30 sec. In contrast, in the presence of substrate, XylE fulfills substrate transport (through a full conformational cycle) in a significantly faster manner. As reported by Davis EO and colleagues[4], xylose transport activity is ~10 nmol D-xylose/mg cells/min. Assuming that the expression level is at most ~5 g cells/mg XylE (~50 kiloDalton) which is the typical yield we have in nutrition-rich LB medium, the rate of xylose influx per protein is ~40 $s^{-1}$. Thus, the average time interval for each xylose transport is about 0.025 sec, which is shorter than our estimate (for *apo* XylE_H system) by three orders of magnitude. In this respect, we think that the 5 kcal/mol free energy could prevent effective structure transition for the protonated *apo* XylE, thus prohibiting proton leak.

## Interpretation of PEGylation assays.

We have calculated pKa values of D27 (XylE_H system) using PROPKA3.1[5,6], and the results are 6.12±0.76 and 4.41±0.86 for IF and OF states, respectively. Obviously, the side-chain of D27 is likely to be deprotonated at pH 7.5 for both conformations. According to our PMF calculation on the XylE_noH system, the *apo* XylE is supposed to reside almost exclusively in the OF state. Unexpectedly, we observed positive PEG labeling in both L65C and V412C mutants, which implied presence of both OF and IF states. We think that the inconsistence arises from the unwanted perturbation on protein thermodynamics induced by Cys mutations required for PEGylation.

Despite the deficiency, the PEGylation assays could still provide sufficient information for the alteration of conformational preference induced by an individual mutation, if attention is focused on the relative experimental values of the protein with this mutation against the one without this mutation. For instance, to elucidate the effect of D27N on the ratio change of population, the only meaningful way of interpretation would be to compare the PEG labeling of D27N+L65C to that of WT+L65C but not D27N+V412C. We then concluded that D27N has a reduced OF state preference comparing to WT, which is consistent with our PMF calculations. The labeling amount of D27N+L65C alone reveals not much insight of the conformation preference of D27N because of the perturbation by L65C.

## XylE D27N mutant is not an effective uniporter.

First of all, we would like to explain the differences between cell-based influx assays and liposome-based counterflow assays. As presented in S11A Fig, there are two types of transport cycles that could generate a net influx of radioactive substrate: (1) uptake of a hot substrate; (2) exchange a hot substrate for an internal cold one. Both cycles are feasible in counterflow assays while only Cycle 1 is possible for cell-based uptake assays. Because we reconstructed proteoliposomes with high sugar concentration (20 mM) inside, the transporter would most likely undergo Cycle 2 for hot substrate accumulation. As of D27N mutant, having counterflow activity but not cell-based influx suggests that the mutant adopts Cycle 2 rather than Cycle 1. This means the IF-OF transition of *apo* D27N (reaction highlighted by red arrow in S11A Fig) is suppressed, which agrees with our free energy calculation of XylE_H. Given the above discussion, we believe that although D27N retains counterflow activity, it is not an effective uniporter with facilitative substrate influx.

## Reference

1. Schurmann A, Doege H, Ohnimus H, Monser V, Buchs A, et al. (1997) Role of conserved arginine and glutamate residues on the cytosolic surface of glucose transporters for transporter function. Biochemistry 36: 12897-12902.
2. Moradi M, Tajkhorshid E (2014) Computational Recipe for Efficient Description of Large-Scale Conformational Changes in Biomolecular Systems. Journal of Chemical Theory and Computation 10: 2866-2880.
3. Appleman JR, Lienhard GE (1985) Rapid kinetics of the glucose transporter from human erythrocytes. Detection and measurement of a half-turnover of the purified transporter. J Biol Chem 260: 4575-4578.
4. Davis EO, Jonesmortimer MC, Henderson PJF (1984) Location of a Structural Gene for Xylose-H+ Symport at 91 Min on the Linkage Map of Escherichia-Coli-K12. Journal of Biological Chemistry 259: 1520-1525.
5. Olsson MH, Sondergaard CR, Rostkowski M, Jensen JH (2011) PROPKA3: Consistent Treatment of Internal and Surface Residues in Empirical pKa Predictions. Journal of Chemical Theory and Computation 7: 525-537.
6. Sondergaard CR, Olsson MH, Rostkowski M, Jensen JH (2011) Improved Treatment of Ligands and Coupling Effects in Empirical Calculation and Rationalization of pKa Values. Journal of Chemical Theory and Computation 7: 2284-2295.
