# Supplementary figures and images for: Molecular determinants for the thermodynamic and functional divergence of uniporter GLUT1 and proton symporter XylE
Source: PLoS Comput Biol. 2017 Jun 15;13(6):e1005603. doi: 10.1371/journal.pcbi.1005603 (PMC5491310; doi:10.1371/journal.pcbi.1005603)

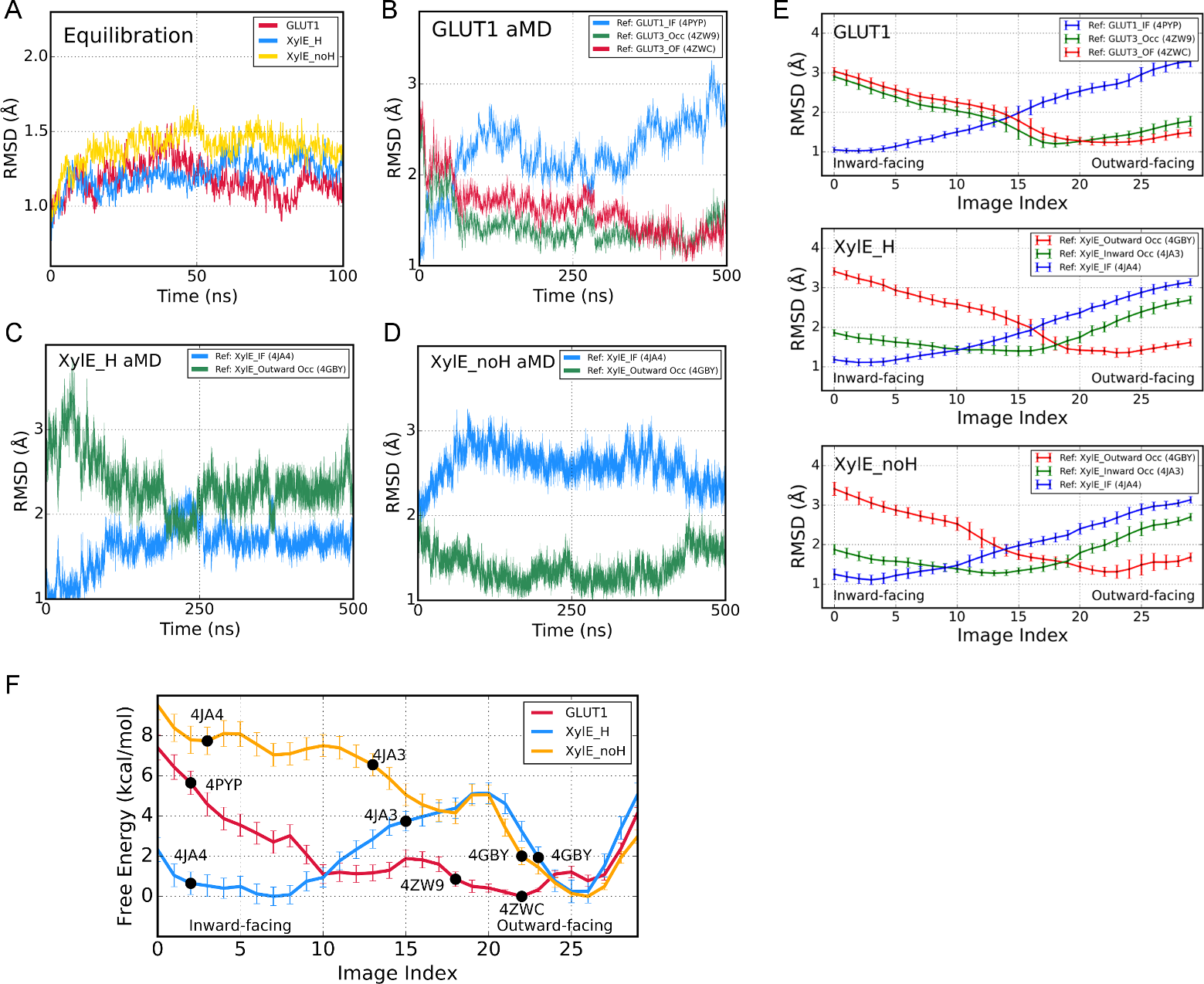

Supplement: S1 Fig — (A) TM-domain Cα RMSD of cMD simulations (Sim #1, #2 and #3) in comparison with the initial structures. The trajectories of GLUT1, XylE_H and XylE_noH are colored red, blue and yellow respectively. The steady RMSD time-series with fairly low values (< 1.5 Å) indicate system stability and modest conformational shift for the 3 systems. (B-D) TM-domain Cα RMSD of aMD simulations (Sim #4, #5 and #6) in comparison with reported crystal structures. GLUT1 (D) transits from IF to OF state, since its RMSD with respect to the outward-facing GLUT3 (PDBID: 4ZWC) structure nearly monotonously drops with time. XylE_H (C) and XylE_noH (D) systems show strong preference for IF and OF conformations respectively, since they mainly sample in the RMSD-based spaces around either inward-open (PDB ID: 4JA4) or outward-occluded (PDB ID: 4GBY) structure of XylE. (E) TM-domain Cα RMSDs of all structure in each window were calculated for the estimation of mean value and standard deviation. The RMSD profiles along the transition path suggest that all 3 systems sequentially visit the IF, Occ and OF conformations. It is worth mentioning that structures in windows near the end of the path could be more widely open than the OF crystal structures (PDB IDs: 4ZWC and 4GBY). (F) PMF profiles with windows of highest structural similarity to known crystal structures labeled as dots. (TIF) [file pcbi.1005603.s002.tif]

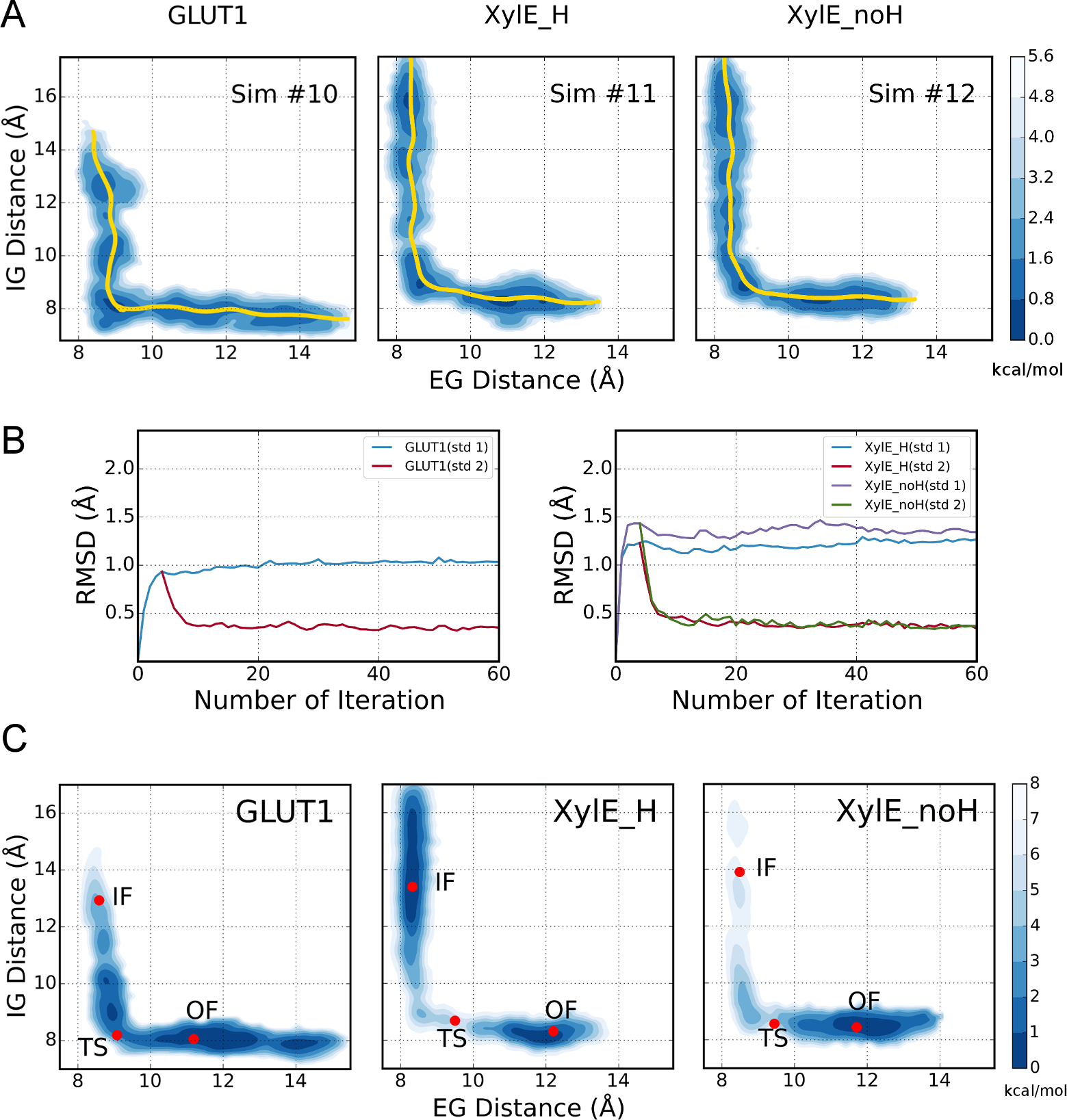

Supplement: S2 Fig — (A) Unweighted contour maps in the space of gate distances, constructed from the equilibrium simulations (Sim #10, #11 or #12). The yellow lines represent the transition paths reconstructed in the space of gate distances, based on equilibrium simulations (see Free energy calculations in Methods section for details). (B) Convergence analysis for the SMwST simulations, using std 1 and std 2 as defined in Methods. (C) PMF profiles in the 2D space of gate distances. The positions of IF, TS and OF states (see S3C Fig for definition) are shown as red dots. (TIF) [file pcbi.1005603.s003.tif]

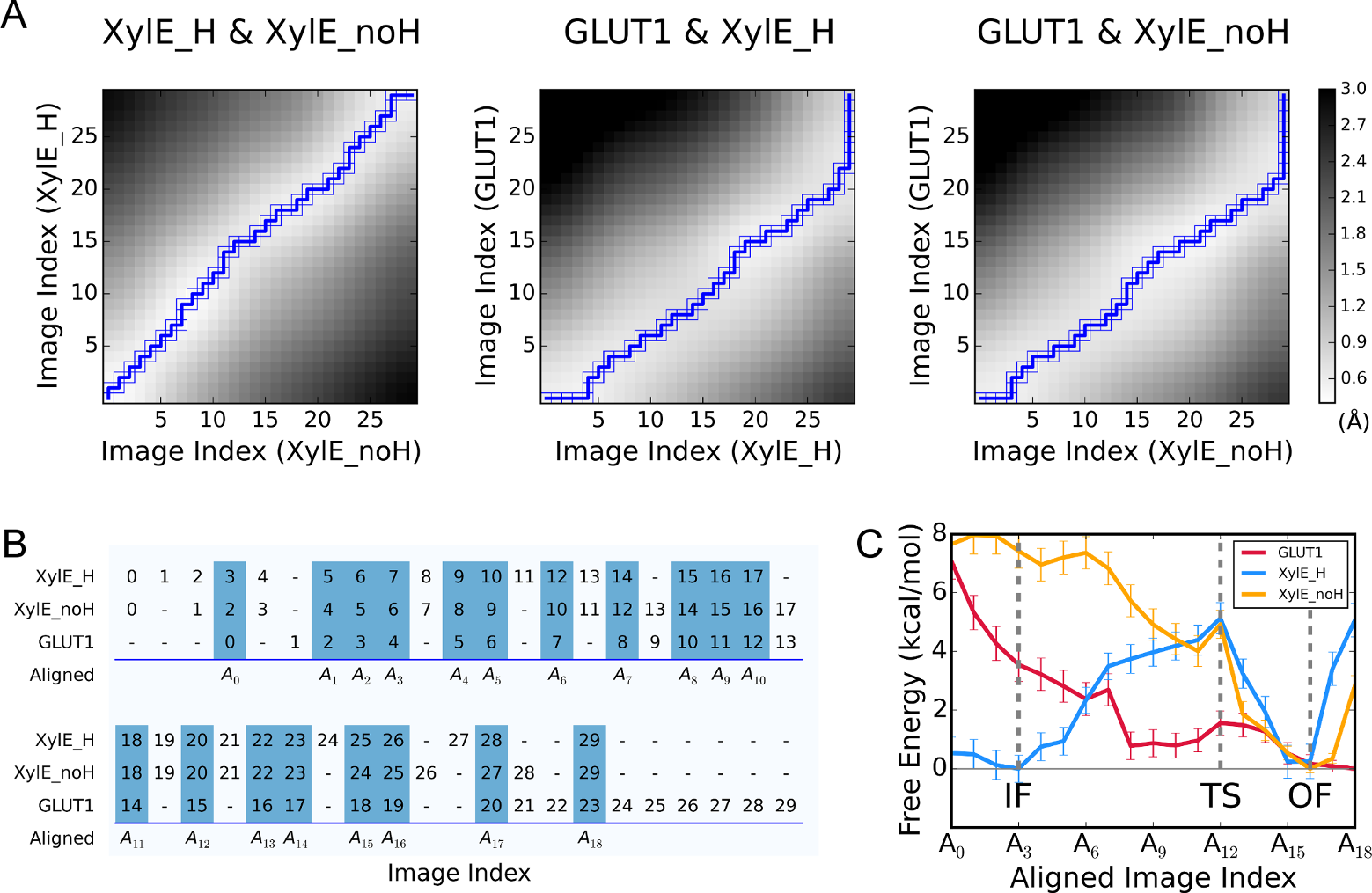

Supplement: S3 Fig — (A) RMSD-based scoring matrix of pairwise path alignment. Average structures of each BEUS window are used to generate a discretized pathway of certain systems. After computing the RMSDs of TM-domain Cα atoms between corresponding representative structures on two paths, we could define a route with minimal summation of RMSDs as aligned. (B) Multi-path alignment of GLUT1, XylE_H and XylE_noH systems. Multi-path alignment is constructed by pairwise alignment results, requiring that only those window triplets bearing lowest summation of RMSD are considered as aligned (see Window alignment in Methods section for details). In total, 19 aligned window triplets were obtained and they were re-numbered. (C) PMF profiles of the aligned windows. Once the BEUS windows are aligned, we can define IF, TS and OF states clearly, as aligned window A3, A12 and A16 respectively, by referring to the free energy profiles. (TIF) [file pcbi.1005603.s004.tif]

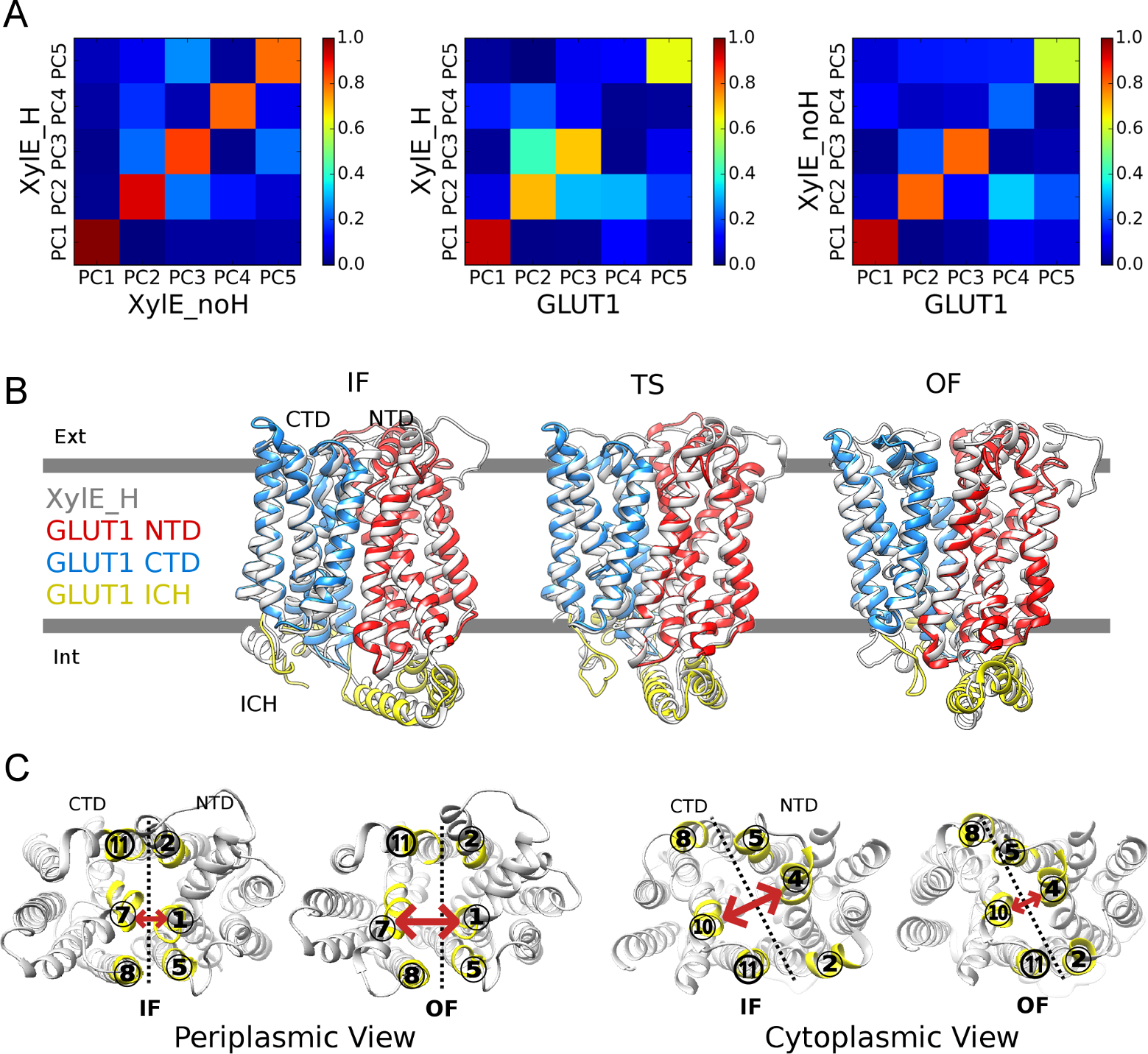

Supplement: S4 Fig — (A) Correlations between top 5 principal components (PCs) of the 3 systems. (B) Visualization of GLUT1 and XylE_H in distinct conformations. Noticeably, rocker-switch motion of NTD and CTD takes place for both systems (also see S1 Movie). (C) Conformational changes of TM helices during the extracellular (periplasmic) and intracellular (cytoplasmic) gating. Red arrows indicate the significant changes in distance between TM helices during gating. The dashed lines show the interface between NTD and CTD. (TIF) [file pcbi.1005603.s005.tif]

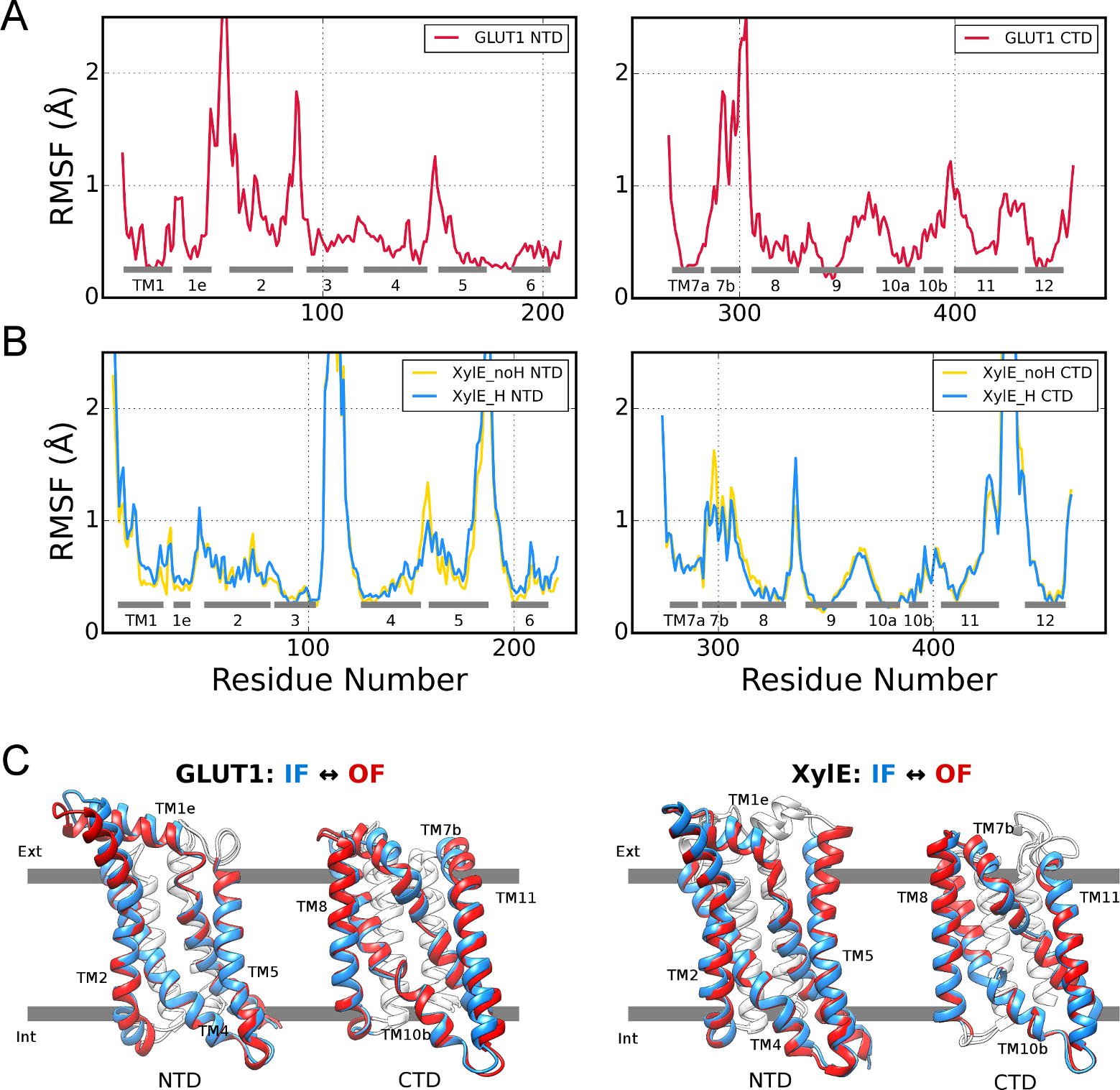

Supplement: S5 Fig — (A-B) RMSFs for the average structures of all aligned BEUS windows. The structures were superimposed upon NTD or CTD before the calculation. Large RMSF values indicate significant local conformational change along the transition path. Regions fluctuating vigorously include numerous loops as well as TM7b. (C) Structure alignment of NTD and CTD in IF (blue) and OF (red) states for GLUT1 and XylE_H. TM helices in the back are uncolored for clarity. The resemblance between IF and OF states supports nearly rigid-body movement. (TIF) [file pcbi.1005603.s006.tif]

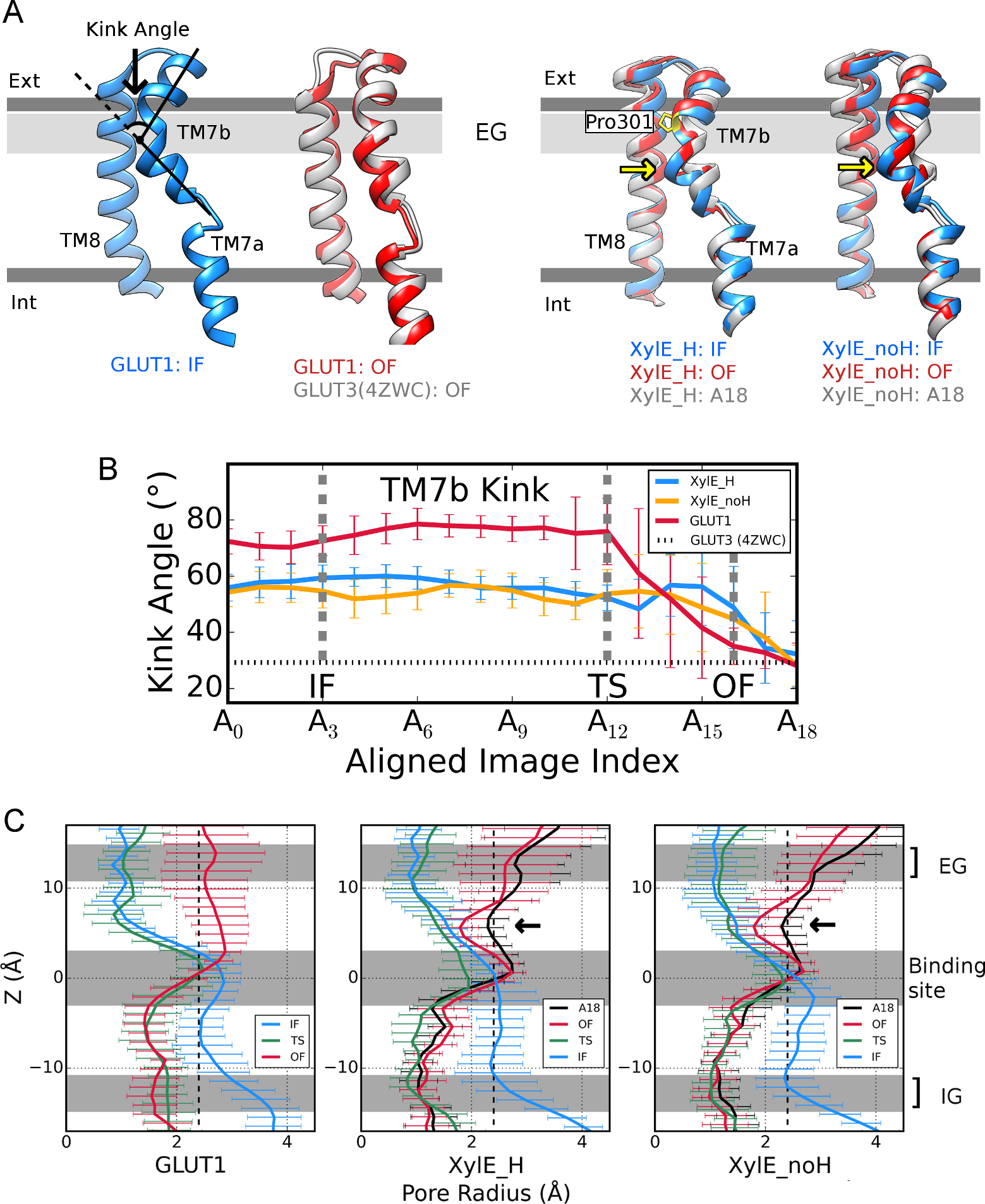

Supplement: S6 Fig — (A) (Left panel) TM7b kinking in the representative structures of IF (blue) and OF (red) states for GLUT1. Definition of the kinking angle is illustrated on the IF structure. OF state of GLUT3 crystal structure is shown in gray for comparison. (Right panel) TM7b kinking in the representative structures of IF (blue) and OF (red) states for XylE_H (left) and XylE_noH (right). Representative structure of the aligned window A18 is shown in gray for comparison. The yellow arrow labels the place where the kinking begins to diminish in window A18. Pro301 that is supposed to facilitate kinking is shown as yellow sticks in the IF structure. (B) The kinking angle of TM7b as illustrated in (A) along the aligned transition paths of GLUT1 (red), XylE_H (blue) and XylE_noH (yellow). The kinking angle in the OF state of GLUT3 crystal structure is shown as black dashed line for reference. (C) Profiles of pore radii along z-axis in the IF (blue), TS (green) and OF (red) states. The vertical dashed lines represent the minimal radius to allow substrate (D-glucose/D-xylose in chair conformation) access. In XylE systems, pore radii of window A18 (see S3C Fig for definition) are also appended (black). Regions of the Extracellular Gate (EG), Intracellular Gate (IG) and substrate binding sites are shown in gray shadow. The black arrow denotes the unique gate identified in the XylE systems. (TIF) [file pcbi.1005603.s007.tif]

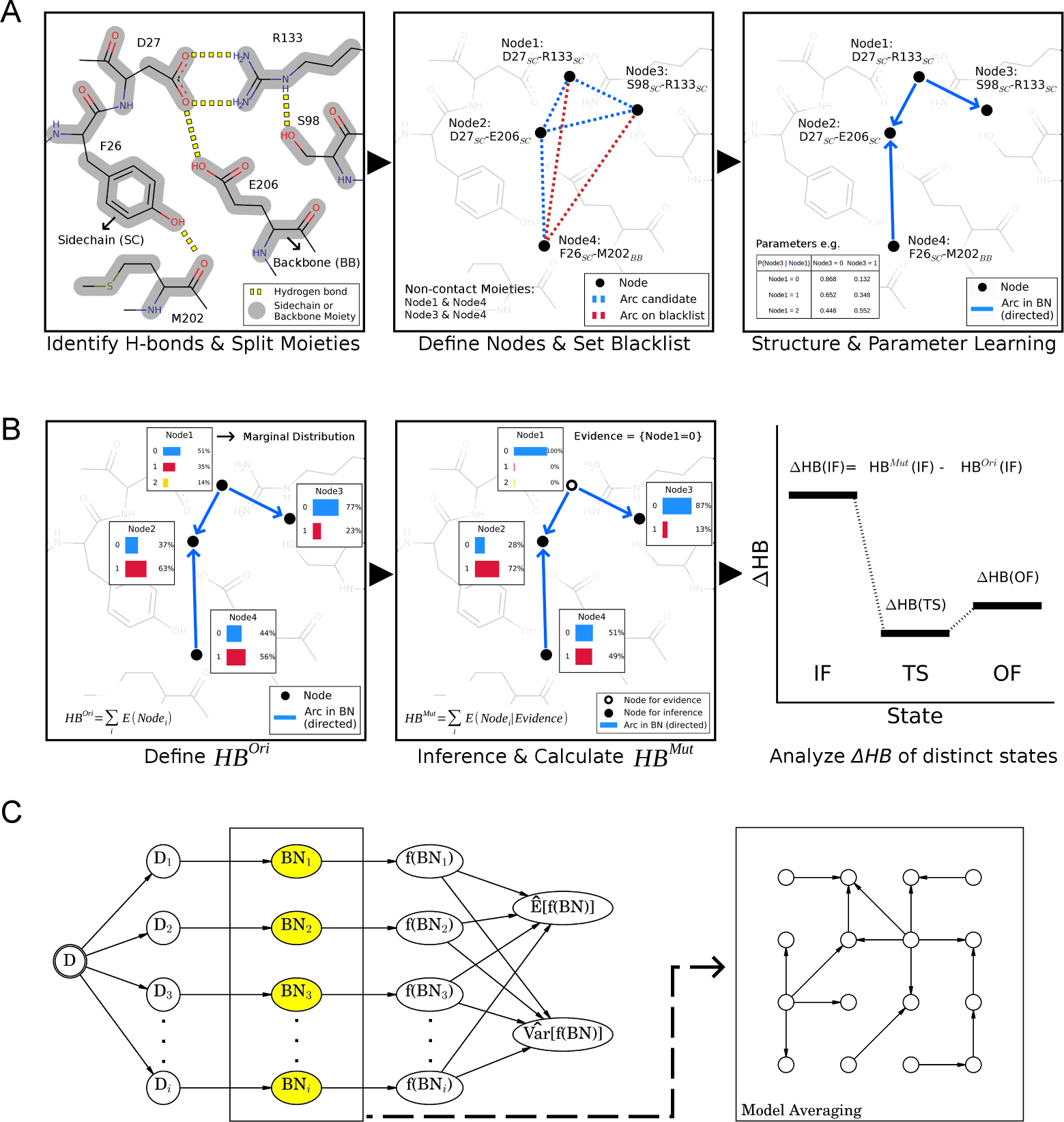

Supplement: S7 Fig — (A) Structure and parameter learning of Bayesian network modeling. (Left panel) Given an aligned BEUS window, we identified the non-trivial H-bonds, which were then combined into side-chain and backbone moieties. (Middle panel) Non-trivial H-bonds were first symbolized as nodes. An arc blacklist was then generated that included the node pairs whose moiety never formed physical contacts in the BEUS simulations. (Right panel) Through training on the dataset (structures in the BEUS window), the structure of the network was established with directed arcs showing causal relationships (or dependencies), and the conditional probability for each directed arc was estimated. (B) Bayesian network inference based on hard evidence. (Left panel) The marginal distributions for each node were used to calculate the expected value of H-bond number for each node. The total amount of H-bonds in the original network, denoted as HBOri, was derived by summing over nodes in the network. (Middle panel) With a hard evidence (e.g., Asp27SC-Arg133SC = 0), the marginal distributions of some random variables change, which therefore describes effect of specific mutations. The conditional expected value of each node conditioned on this evidence was calculated. The total amount of H-bonds in the perturbed network, denoted as HBMut, was then derived by summing over nodes. (Right panel) Based on the Bayesian network model constructed from the corresponding BEUS window, ΔHB = HBMut–HBOri can be calculated for one state, which quantitatively evaluates the overall H-bond change. Finally, ΔΔHB is derived to evaluate the influence of specific mutations on the transition between two states (see Fig 3B for details). (C) (Left panel) Estimation of a Bayesian network (BN) feature denoted as f(BN) by cross-validation (HB in our case). Mean and standard deviation could be easily derived from the models learned from resampled data. (Right panel) These Bayesian network (BN) models were averaged to generate a robu [file pcbi.1005603.s008.tif]

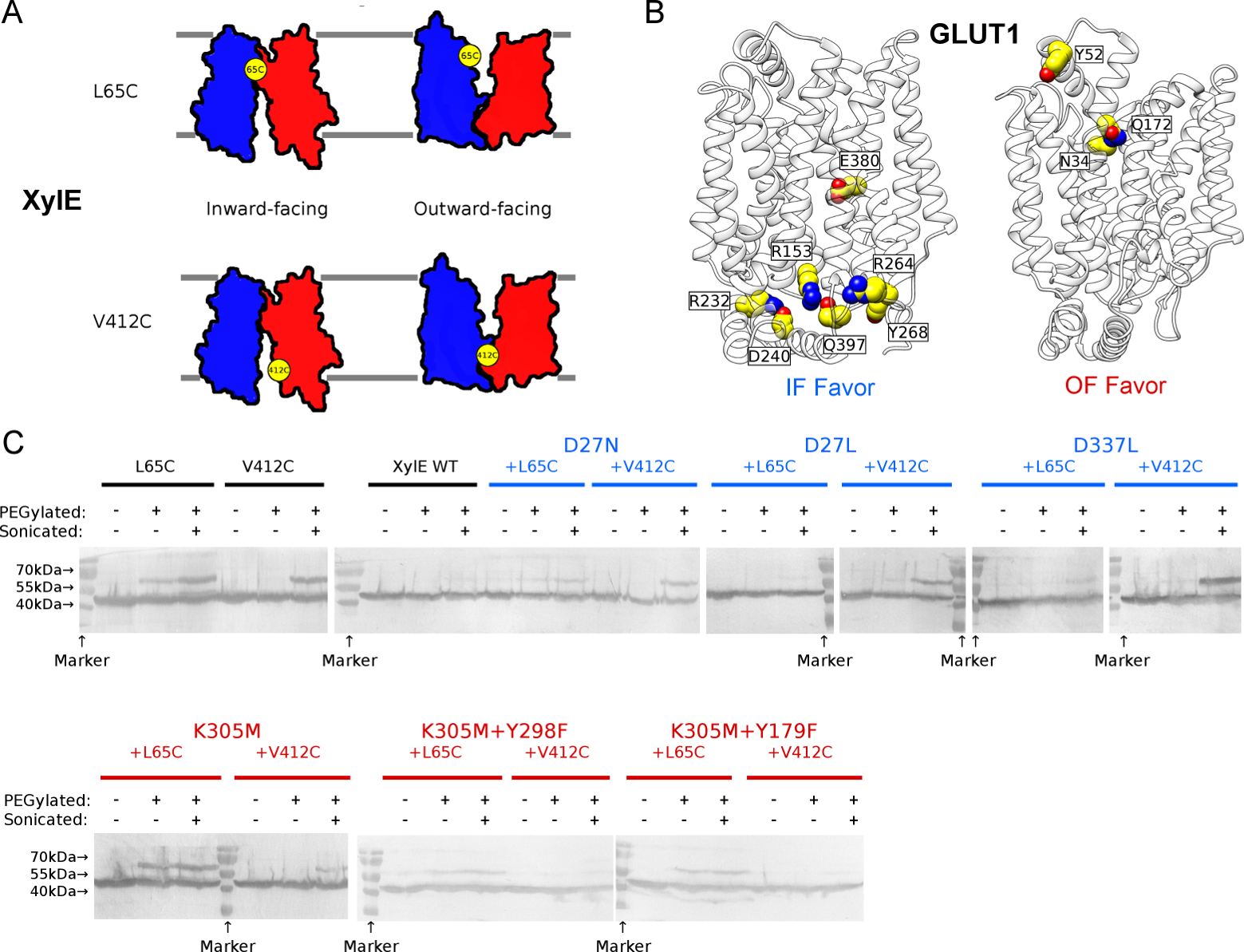

Supplement: S8 Fig — (A) Solvent accessibility of the PEGylation sites L65C and V412C at certain states. L65C and V412C can only be labeled by mPEG from periplasmic and cytoplasmic sides respectively. (B) Predicted GLUT1 side-chain mutations that strongly prefer IF and OF states. The residues favoring OF and IF should satisfy the individual condition of ΔΔHBOF→IF < -0.7 and ΔΔHBOF→IF > 0.7 respectively (see Fig 3C for details). (C) Western blot results of PEGylation assays. Control groups indicate that WT XylE has no available cysteines for labeling, while L65C and V412C can be modified without and with sonication step to break the cells respectively. Mutants favoring IF and OF conformations are colored blue and red respectively. (TIF) [file pcbi.1005603.s009.tif]

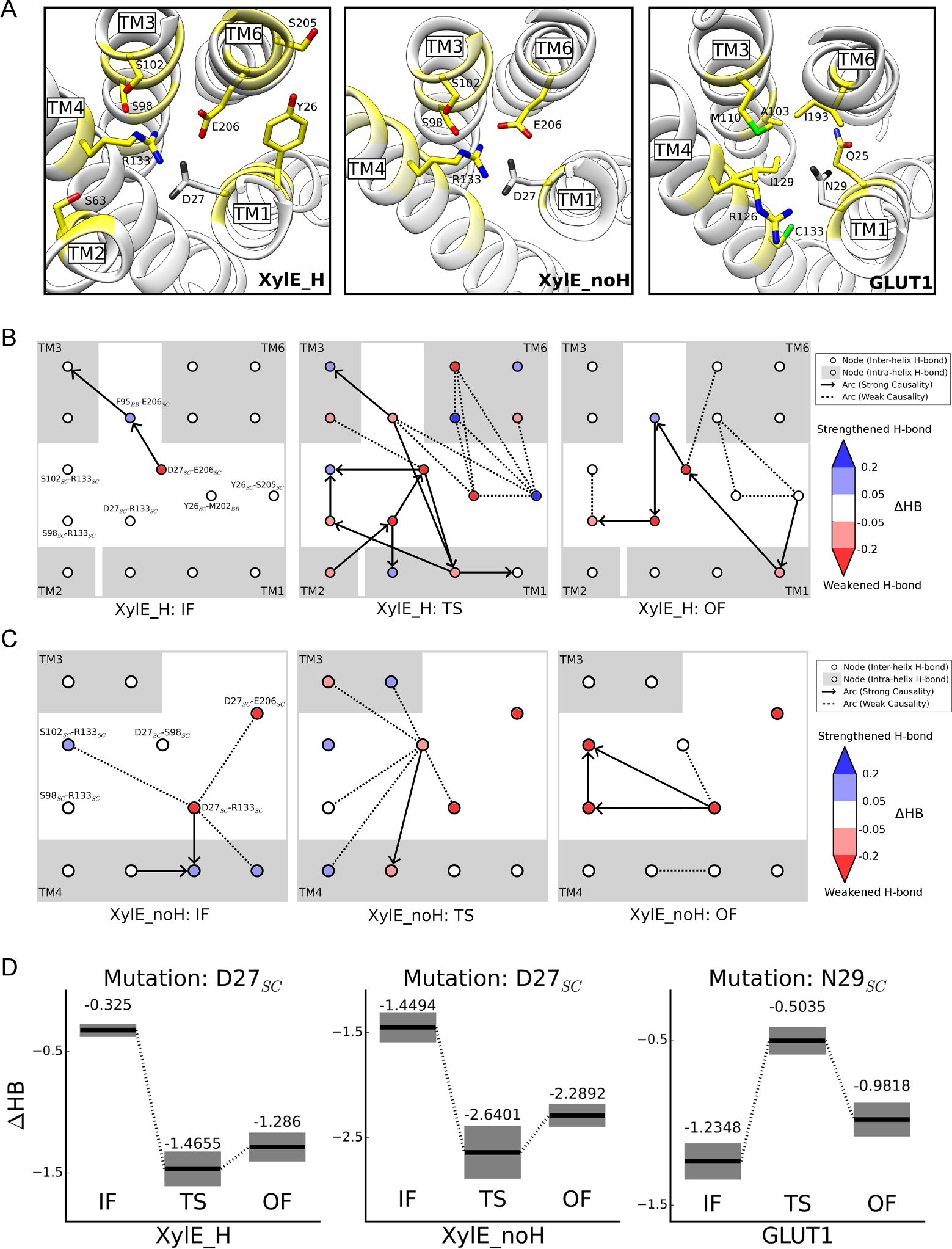

Supplement: S9 Fig — (A) Structural views of the local network around Asp27/Asn29 inside NTD. In each system, the residues affected by network intervention based on Bayesian network inference are colored yellow. Asp27SC and Asn29SC are decolorized to emphasize the removal of all relevant interactions. (B-C) Detailed variations of influenced local networks in XylE systems. Similar to Fig 4C and 4D, the robust Bayesian network models were generated by model averaging (see S7C Fig). (D) ΔHB profiles in IF, TS and OF states for the 3 systems. The error bars in gray represent the standard deviations that were calculated by resampling technique (see S7C Fig). (TIF) [file pcbi.1005603.s010.tif]

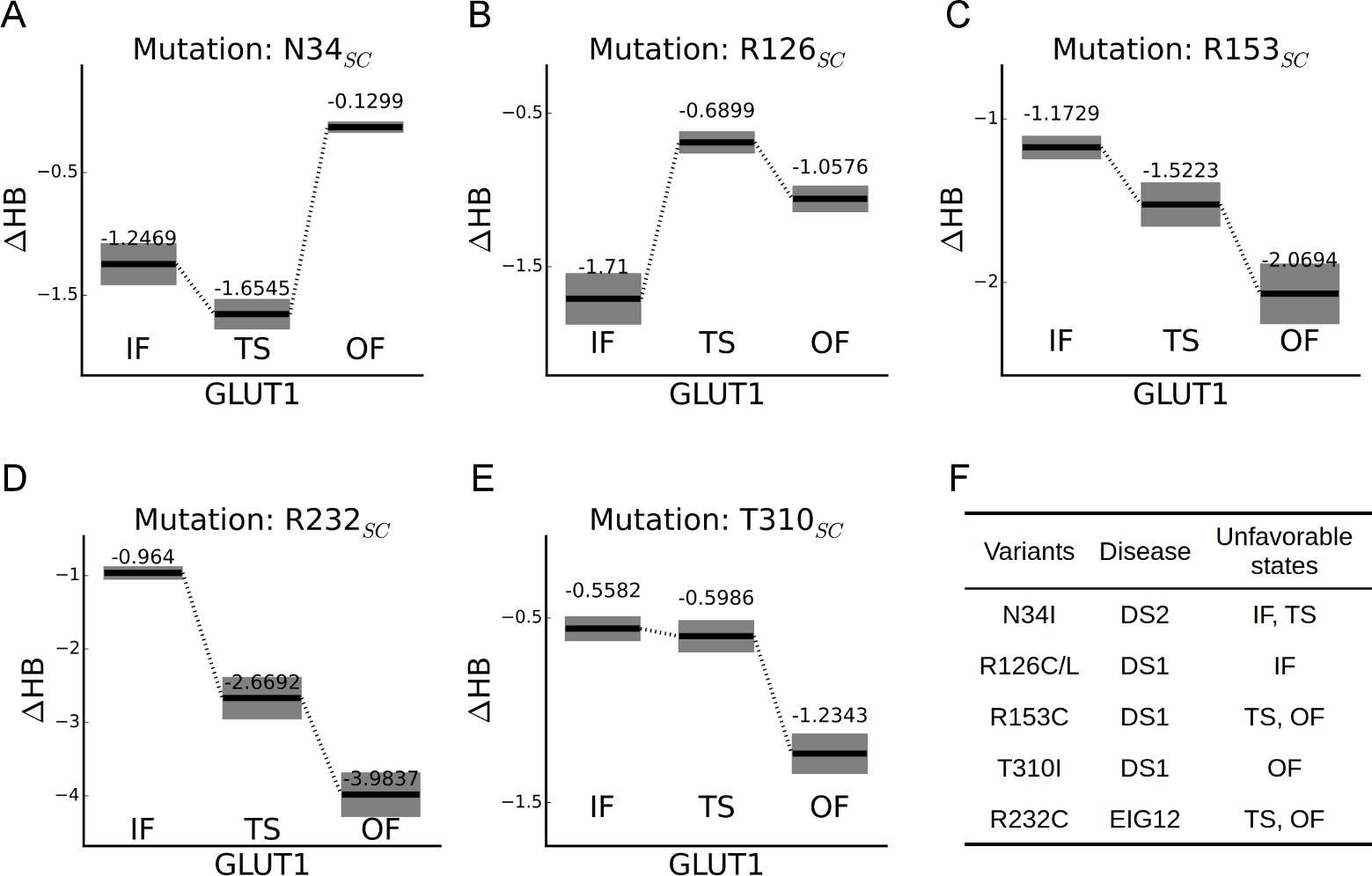

Supplement: S10 Fig — (A-E) ΔHB profiles of disease-related mutations. In all mutations, the polar/charged residues are substituted by hydrophobic ones, which therefore disable side-chains H-bonds. According to Bayesian network inference, all of these mutations substantially alter the energetic balance between IF and OF states, thereby presumably devastating the transport activity of GLUT1. (F) Summary of the variants. The listed mutations were discovered to cause ‘GLUT1 deficiency syndrome 1/2’ (DS1/2) or ‘epilepsy, idiopathic generalized 12’ (EIG12). (TIF) [file pcbi.1005603.s011.tif]

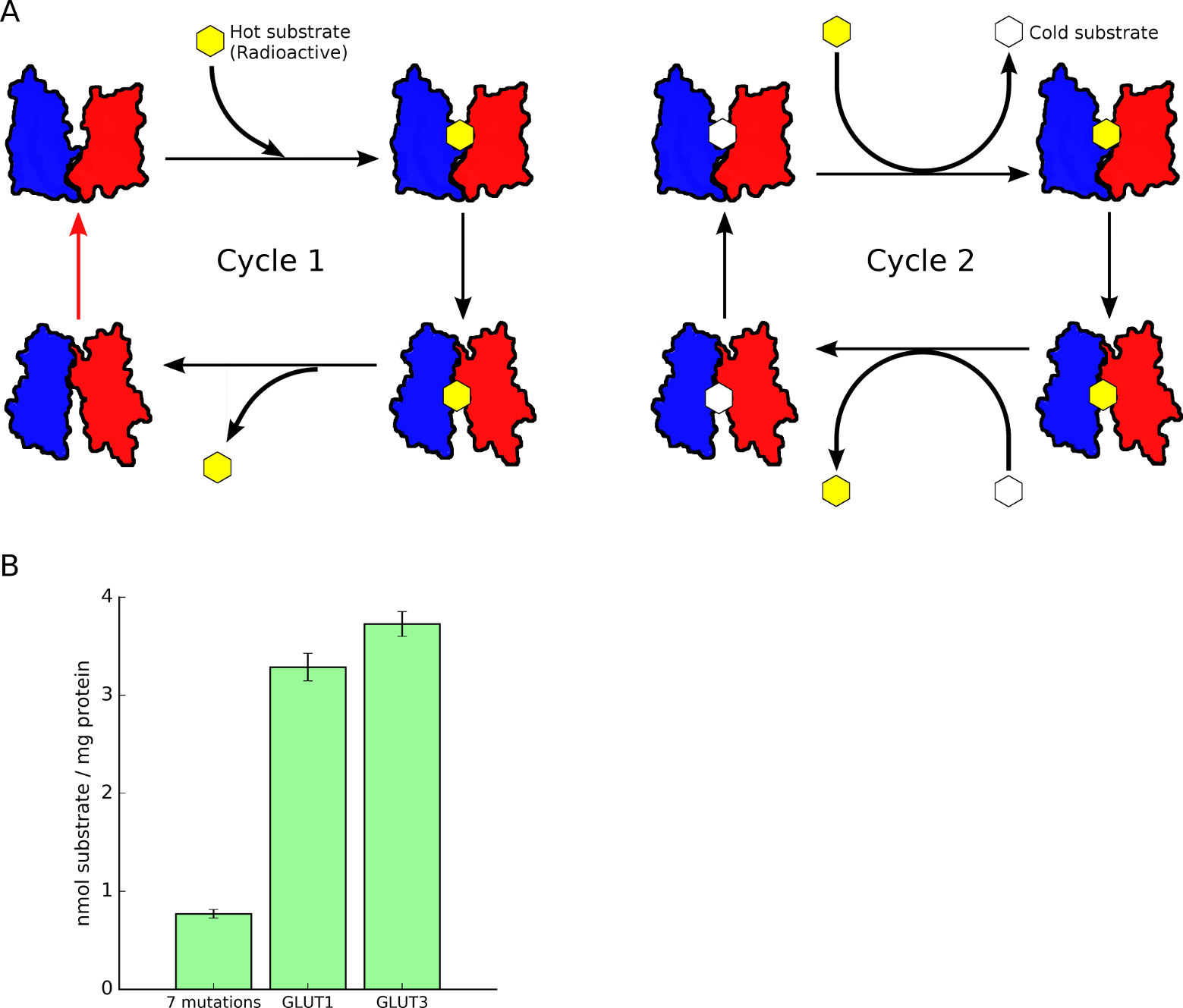

Supplement: S11 Fig — (A) Transport cycles that could generate a net influx of radioactive substrate. (Left panel) Uptake of a hot substrate. (Right panel) Exchange a hot substrate for an internal cold one. (B) Counterflow activities of XylE 7-mutation variant, GLUT1 and GLUT3. The negative control has already been subtracted. (TIF) [file pcbi.1005603.s012.tif]
